# RNA cytosine methyltransferase NSUN5 promotes protein synthesis and tumorigenic phenotypes in glioblastoma

**DOI:** 10.1002/1878-0261.13434

**Published:** 2023-04-22

**Authors:** Jiesi Zhou, Yan Shu Kong, Krista M. Vincent, Dylan Dieters‐Castator, Amirali B. Bukhari, Darryl Glubrecht, Rong‐Zong Liu, Douglas Quilty, Scott D. Findlay, Xiaowei Huang, Zhihua Xu, Rui Zhe Yang, Lanyue Zhang, Emily Tang, Gilles Lajoie, David D. Eisenstat, Armin M. Gamper, Richard Fahlman, Roseline Godbout, Lynne‐Marie Postovit, YangXin Fu

**Affiliations:** ^1^ Department of Oncology, Faculty of Medicine and Dentistry University of Alberta Edmonton AB Canada; ^2^ Department of Biochemistry Western University London ON Canada; ^3^ Department of Biomedical and Molecular Sciences Queen's University Kingston ON Canada; ^4^ Department of Paediatrics University of Melbourne Parkville Vic. Australia; ^5^ Department of Biochemistry, Faculty of Medicine and Dentistry University of Alberta Edmonton AB Canada

**Keywords:** glioblastoma, NSUN5, protein synthesis, RNA cytosine methylation

## Abstract

Glioblastoma (GBM) is the most common and aggressive malignant primary brain tumor in adults. The standard treatment achieves a median overall survival for GBM patients of only 15 months. Hence, novel therapies based on an increased understanding of the mechanistic underpinnings of GBM are desperately needed. In this study, we show that elevated expression of 28S rRNA (cytosine‐C(5))‐methyltransferase NSUN5, which methylates cytosine 3782 of 28S rRNA in GBM cells, is strongly associated with the poor survival of GBM patients. Moreover, we demonstrate that overexpression of *NSUN5* increases protein synthesis in GBM cells. *NSUN5* knockdown decreased protein synthesis, cell proliferation, sphere formation, migration, and resistance to temozolomide in GBM cell lines. *NSUN5* knockdown also decreased the number and size of GBM neurospheres *in vitro*. As a corollary, mice harboring U251 tumors wherein *NSUN5* was knocked down survived longer than mice harboring control tumors. Taken together, our results suggest that NSUN5 plays a protumorigenic role in GBM by enabling the enhanced protein synthesis requisite for tumor progression. Accordingly, NSUN5 may be a hitherto unappreciated target for the treatment of GBM.

## Graphical abstract

Elevated NSUN5 in GBM increases methylation of cytosine 3782 of 28S rRNA, which might change the structure and/or activity of ribosome. This consequently causes increased mRNA translation and promotes tumorigenic phenotypes of GBM, including stemness, cell growth, resistance to temozolomide, and migration *in vitro* and *in vivo*, suggesting that NSUN5 can be a potential therapeutic target to treat GBM.

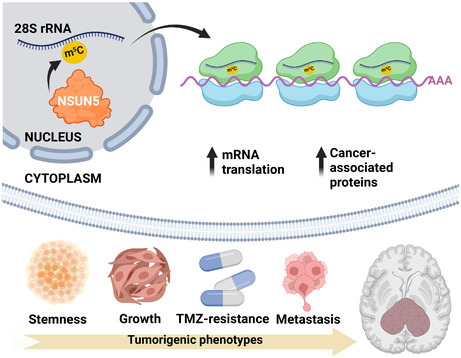

**Abbreviations**
BLI: bioluminescence imaging
CCI: Cross Cancer Institute
GBM: glioblastoma
GO: gene ontology
GSCs: glioblastoma stem cells
IHC: immunohistochemistry
KO: knockout
LC–MS/MS: liquid chromatography–tandem mass spectrometry
MGMT: O^6^‐methylguanine‐DNA methyltransferase
NSG: DOD‐SCID IL2R‐gamma null
RT‐qPCR: reverse transcription‐quantitative PCR
STR: short tandem repeat
TMZ: temozolomide
WT: wild‐type

## Introduction

Glioblastoma (GBM) is the most common and aggressive malignant primary brain tumor in adults [1]. Despite the aggressive standard treatment of maximal surgical resection followed by radiation and chemotherapy, the median overall survival of patients with GBM is approximately 15 months from diagnosis [2, 3]. The infiltrative growth, genetic and cellular heterogeneity, and presence of tumorigenic cancer stem cells account for failure of the treatment and recurrence of GBM, highlighting the need to better understand the molecular mechanisms of the disease to develop novel therapeutics, including targeted therapies [4]. Glioblastoma stem cells (GSCs) promote intratumoral heterogeneity and resistance to treatment, rendering them a crucial therapeutic target, which necessitates deeper understanding of the plasticity and resistance mechanisms of GSCs [5, 6]. Epigenetic mechanisms are also involved in GBM. For examples, silencing of the DNA repair gene O^6^‐methylguanine‐DNA methyltransferase (MGMT) due to promoter DNA methylation sensitizes GBM tumors to temozolomide (TMZ, an alkylating agent) and MGMT promoter methylation is a prognostic marker for a favorable outcome in GBM patients treated with TMZ [7]. Furthermore, emerging evidence suggests that RNA modifications, such as N^6^‐methyladenosine (m^6^A), play an important role in GBM tumorigenesis [8, 9]. However, the role of RNA cytosine methylation in GBM remains elusive.

RNA modifications at selective sites regulate the biology and functions of all species of RNAs. These modifications govern processes such as splicing, nuclear export, stability, and mRNA translation. RNA modifications also affect the expression, structure and functions of noncoding RNAs, including transfer (t)RNAs, ribosomal (r)RNAs, microRNAs and long noncoding RNAs (lncRNAs) [10]. Dysregulation of RNA modifications caused by the aberrant expression or mutations of RNA modifiers alters the epitranscriptome, which is implicated in multiple processes of cancer development, such as initiation, progression, stemness, and resistance to therapy [11]. Thus far, more than 100 modifications catalyzed by specific enzymes have been identified in eukaryotic RNAs, including dozens of methylations in ribonucleosides, such as m^6^A and 5‐methylcytosine (m^5^C) [12]. m^5^C, which is present in diverse RNA species, is mediated by a family of Nol1/Nop2/SUN domain (NSUN) RNA cytosine methyltransferases and DNA methyltransferase homolog DNMT2, which is also known as TRDMT1 [13]. There are seven NSUN proteins (NSUN1‐7) in humans that methylate different RNA targets [13]. The functional impact of m5C depends on the species of the target RNAs and the sites of methylation. Although regulation and function of m^5^C in RNAs remain largely elusive, a role for m^5^C in cancer is emerging [11].

NSUN5 has been identified as an RNA methyltransferase for rRNA. In yeast, Rcm1 (the yeast homolog of NSUN5) methylates cytosine 2278 within a conserved region of 25S rRNA [14, 15]. Loss of Rcm1 results in a structural change in 25S rRNA, which in turn alters ribosomal conformation concomitant with translational reprogramming, leading to the selective translation of a subset of oxidative stress‐responsive mRNAs, resistance to stress and increased life span [14], indicating that alterations in NSUN5‐dependent rRNA methylation have a significant biological consequence [14, 15]. Thus far, knowledge of the biological functions of NSUN5 in mammals is still limited. In mouse, knockout of the *Nsun5* gene did not affect postnatal growth and energy metabolism, but impaired development and function of oligodendrocyte precursor cells and was associated with cognitive deficits [16]. The same group also showed that Nsun5 plays a critical role in the development of the cerebral cortex and knockout of the *Nsun5* gene led to agenesis and hypomyelination of the corpus callosum [17, 18]. The human *NSUN5* gene is located on chromosome 7 and is completely deleted in the Williams–Beuren syndrome, a complex neurodevelopmental disorder [19]. Thus far, only three publications have addressed the role of NSUN5 in cancer [20, 21, 22]. Jiang et al. showed that high expression of NSUN5 promotes cell proliferation in colorectal cancer [22] and Heissenberger et al. showed that *NSUN5* knockout decreases proliferation and the size of HeLa cells, a cervical cancer cell line [20]. However, Janin et al. reported that NSUN5 inhibits cell proliferation and tumor progression in GBM [21]. Therefore, further studies are warranted to determine the role of NSUN5 in cancer to better understand the implication of NSUN5, and RNA cytosine methylations in general, in tumorigenesis.

In this study, we found that elevated NSUN5 expression is associated with shorter overall and progression‐free survival of GBM patients. Our results indicate that NSUN5 methylates cytosine 3782 of 28S rRNA, and promotes protein synthesis and tumorigenic phenotypes in GBM, suggesting that NSUN5 is a potential therapeutic target for GBM.

## Materials and methods

### Analysis of TCGA and CPTAC datasets

The R2 Genomics Analysis and Visualization platform was used to analyze NSUN5 expression as a function of overall survival and progression‐free survival in GBM patients [23]. The Kaplan–Meier Scanner and Tumor GBM‐TCGA‐540 dataset (Affymetrix U133A mRNA expression profiling) were selected for analyzing the association between NSUN5 expression and overall and progression‐free survival of GBM patients. We also analyzed the association of other NSUN family members (where data are available) with overall survival of GBM patients. The cut‐off points for stratifying NSUN5 expression into NSUN5‐low and high subpopulations was determined to be 128.4 and 133.8 for overall and progression‐free survival, respectively, using the scan mode option, which generates the most significant expression cut‐off for survival analysis. We also analyzed the exon expression RNAseq profile of a TCGA Glioblastoma dataset containing 153 patients on the UCSC Xena platform for the expression of all the NSUN family members (NSUN1‐7). For this analysis, we used the median expression value of each gene examined as cut‐off for stratifying high and low expression populations. Additionally, we performed an analysis to compare NSUN5 expression in normal brain (nontumor) and GBM tissues using the TCGA_GBM HG‐U133A dataset on Gliovis platform. Furthermore, the association between NSUN5 protein levels and survival of GBM patients was analyzed using the proteomic dataset on the CPTAC portal as reported by Wang et al. [24].

### Cell culture

Human GBM cell lines U251 (RRID:CVCL_0021), T98 (RRID:CVCL_B368), U87 (RRID:CVCL_0022), and A172 (RRID:CVCL_0131) were obtained from Dr. Roseline Godbout and cultured in DMEM low glucose medium, supplemented with 10% FBS, 100 U·mL^−1^ penicillin, and 100 μg·mL^−1^ streptomycin [25]. These cell lines were authenticated by Short Tandem Repeat (STR) DNA profiling in the past 3 years. For cell authentication, genomic DNA isolated from the cell lines was submitted to the Centre for Applied Genomics at the Hospital for Sick Children (Toronto, Canada) for STR profile testing. The STR profiles were then compared with the published cell identification data online (https://celldive.dsmz.de/str/browse) to confirm the identify of the cells. GBM primary neurosphere cultures A4‐012, ED511 and 50M were established by Dr. Roseline Godbout [26], Dr. Kenneth Petruk, and Dr. Samuel Weiss [27], respectively, and were cultured in DMEM low glucose medium, supplemented with 20 ng·mL^−1^ EGF, 20 ng·mL^−1^ FGF, and 1X B27 supplement. These GBM primary neurosphere cultures were established directly from patient tissues that were confirmed as GBM tumors by neuropathologists and immediately frozen. Institutional approval for research with human materials was received prior to the acquisition of GBM biopsies for this study (Health Research Ethics Board of Alberta Cancer Committee, HREBA. CC‐14‐0070). The study methodologies conformed to the standards set by the Declaration of Helsinki. The experiments involving GBM biopsies were undertaken with the understanding and written consent of each subject. The GBM biopsies were collected at the Cross Cancer Institute (CCI) within last 5–15 years. HEK293T cells were cultured in DMEM high glucose medium, supplemented with 5% FBS, 100 U·mL^−1^ penicillin, and 100 μg·mL^−1^ streptomycin. All experiments were performed with mycoplasma‐free cells.

### RNA isolation and reverse transcription‐quantitative reverse transcription‐PCR (RT‐qPCR)

RNA isolation, reverse transcription (RT), and quantitative RT‐PCR were performed as previously described [28]. PCR primer sequences are listed in Table S1.

### Preparation of whole‐cell lysates and Western blotting

Whole‐cell lysates were prepared using modified radioimmunoprecipitation assay (RIPA) buffer as described previously [28]. The following primary antibodies were used for Western blotting at 1 : 1000 dilution: NSUN5 (H‐10) (Santa Cruz Biotechnology, Dallas, TX, USA; Cat# sc‐376 147, RRID: AB_10989978), NSUN2 (Proteintech, Rosemont, IL, USA; Cat# 20854‐1‐AP, RRID: AB_10693629), STAT3 (124H6) (Cell Signaling Technology, Danverss, MA, USA; Cat#9139, RRID: AB_331757), β‐Actin (Sigma‐Aldrich, Oakville, ON, Canada; Cat# A5441, RRID: AB_476744), and Tubulin (Abcam, Toronto, ON, Canada, ab59680).

### Immunofluorescence staining

NSUN5 expression and intracellular localization of NSUN5 in GBM cells were examined by immunofluorescence as previously described [28]. Fixed cells grown on the coverslips were immunostained with anti‐NSUN5 antibody at a 1 : 100 dilution, followed by Alexa Fluor^®^ 488 anti‐mouse secondary antibodies (Cells Signaling, Danvers, MA, USA) at a 1 : 200 dilution. DAPI staining (1 μg·mL^−1^) was used to detect nuclei. Images were captured using confocal and AMG EVOS FL microscope (Bothell, WA, USA).

### NSUN5 CRISPR/Cas9 knockout

NSUN5 knockout plasmids were generated as previously described [29]. Two NSUN5 CRISPR guide RNAs targeting exon II of the *NSUN5* gene were designed (Table S1). NSUN5 CRISPR knockout plasmids (5 μg) were transfected into U251 cells using Lipofectamine 2000 (Invitrogen, Burlington, ON, Canada). 72 h post‐transfection, the cells were sorted by fluorescence‐activated cell sorting (FACS) for mCherry into 96‐well plates with a single cell per well. Cells were expanded and screened for positive NSUN5 knockout clones by Western blotting. Induction of indels in exon II of the NSUN5 gene in the positive clones was confirmed by Sanger sequencing.

### Generation of NSUN5 knockdown and overexpression cells

NSUN5 in U251, T98, A4‐012, and ED511 cells was knocked down using two lentivirus‐mediated shRNAs (Origene, Rockville, MD, USA; Catalogue: TL302876) (Table S1). A scrambled shRNA (shControl) was used as a negative control. Overexpression of NSUN5 in U87, A172, and 50M cells was obtained by stable transduction of the pLenti‐NSUN5‐Myc‐DDK vector (Qrigene, Catalogue: RC200144L3) or an empty vector (negative control), followed by puromycin selection. Lentivirus was generated in 293T cells and used to infect the target cells as described previously [30]. Stable knockdown and overexpression of NSUN5 were confirmed by Western blotting.

### Bisulfite sequencing

RNA bisulfite sequencing was conducted to determine the methylation status of cytosine 3782 and cytosine 4447 (C3782 and C4447) of 28S rRNA using EZ RNA Methylation Kit (ZYMO Research, Irvine, CA, USA). Bisulfite‐treated RNAs were reversely transcribed into cDNA using random primers. cDNA fragments containing C3782 or C4447 of 28S rRNA were amplified by PCR using bisulfite sequencing primers (Table S1) and cloned into TOPO vector (TOPO TA Cloning Kit, Thermo Fisher, Mississauga, ON, Canada) for Sanger sequencing. Sequencing results were compared with the original sequences of 28S rRNA using the MultAlin online platform to identify the cytosine methylation sites.

### Puromycin labeling assay

GBM cells were treated with 10 μg·mL^−1^ Puromycin Hydrochloride (Sigma) for 10 min. Cell lysates were collected and applied to Western blotting using puromycin detection via antipuromycin antibody (clone 12D10, Millipore, Oakville, ON, USA; Cat# MABE343, RRID:AB_2566826) to identify the nascent peptide chains containing puromycin [31]. Signal of the puromycin incorporated proteins was first normalized to β‐actin or β‐tubulin loading controls and then standardized against the shControl or the empty vector control.

### Proteomic analysis of NSUN5‐regulated proteins

Cell lysates collected from U251 cells with or without NSUN5 knockdown and 50M cells with or without NSUN5 overexpression in three replicates were processed and analyzed using liquid chromatography–tandem mass spectrometry (LC–MS/MS) as we previously described [32]. Briefly, after chloroform/methanol precipitation and on‐pellet in‐solution digestion, the peptides were analyzed using an M‐class nanoAquity UPLC system (Waters) connected to a Q Exactive mass spectrometer (Thermo Scientific). Peptides were loaded onto an ACQUITY UPLC M‐Class Symmetry C18 Trap Column (5 μm, 180 m × 20 mm) and trapped for 5 min at a flow rate of 10 μL·min^−1^ at 99% solution A (A: Water/0.1% formic acid)/1% solution B (B: Acetonitrile/0.1% formic acid) and subsequently separated on an ACQUITY UPLC M‐Class Peptide BEH C18 Column (130 A, 1.7 μm, 75 μm × 250 mm) operating at a flow rate of 300 nL·min^−1^ at 35 °C using a nonlinear gradient consisting of 1–7% B over 1 min, 7–23% B over 179 min, 23–35% B over 60 min before increasing to 95% B and washing. Settings for data acquisition on the Q Exactive are outlined in Table S2. MS files were searched in maxquant (1.5.8.3) using the Human UniProt database (updated May 2017 with 42 183 entries; refs. [33, 34]). The differentially expressed proteins were screened using the Student's *t*‐test (*P* < 0.05). The raw data of the proteomic analysis for U251 and 50M cells are provided in Tables S3 and S4, respectively. For the label Free Quantification Proteomic Analysis, the ion intensities for the proteins identified between experimental groups were statistically compared with a two‐tailed homoscedastic *T*‐test. Proteins that were identified to be statistically (*P* < 0.05) upregulated or downregulated were analyzed for Gene Ontology (GO) enrichment using STRING database web interface [35]. Cluster alignment of the data was performed with clustergrammer [36]. Prior to statistical comparisons of the proteomic data, missing values were imputed with half local minima values and the ion intensities were corrected as a fraction of the total ion current for each sample. The relative ion intensities for the proteins identified between experimental groups were statistically compared with an unpaired two‐tailed homoscedastic *T*‐test. To minimize type I statistical errors as a result of multiple testing, the criteria of > 2‐fold change and a *P*‐value of < 0.05 were applied [37].

### Neutral red uptake assay

The neutral red uptake assay was used to determine the number of viable cells as previously described [38]. Briefly, cells were seeded into 96‐well plates at a density of 500 cells per well, and cell numbers were measured on Days 1 and 5. Cell proliferation was expressed as the fold change relative to Day 1. To determine the growth of patient‐derived neurospheres, cells were seeded into ultra‐low attachment 96‐well plates at a density of 100 to 500 cells per well, and cell numbers were measured on Day 10 using the modified neutral red assay as previously described [38]. Cell proliferation was expressed as fold change relative to the empty‐vector or shRNA controls. To determine the cytotoxicity of Temozolomide (TMZ), cells were seeded into 96‐well plates at a density of 500 cells per well, treated with increasing concentrations of TMZ for 96 h (0, 62.5, 125, 250, 500 μm for U251 cells; 0, 250, 500, 750, 1000 μm for T98 cells), and subjected to the neutral red uptake assay. Because U251 cells are more sensitive to TMZ than T98 cells, we used lower concentrations of TMZ to treat U251 cells compared with T98 cells. The concentration of TMZ stock was 100 mm dissolved in DMSO. Cell viability was expressed as the percentage relative to the untreated samples.

### Clonogenic assay

The clonogenic assay was conducted as previously described [30]. Briefly, cells were seeded into six‐well plates at a density ranging from 100 or 200 cells per well (untreated controls) to 1600 or 3200 cells per well (treatment with the highest concentration of TMZ). The cells were allowed to attach overnight and were treated with increasing concentrations of TMZ for 72 h (0, 6.25, 12.5, 25, 50, 100 μm for U251 cells; 0, 62.5, 125, 250, 500, 1000 μm for T98 cells). We used lower concentrations of TMZ for the clonogenic assay than for the neutral red uptake assay for U251 cells because cells seeded for the clonogenic assay are more sensitive to TMZ than for the neutral red assay. The medium with TMZ was then replaced with fresh medium to allow the cells to grow into colonies (approximately 10–14 days). The colonies were fixed with 4% paraformaldehyde, stained with crystal violet (0.5% in methanol), and counted to calculate cell viability as previously described [30].

### Migration assay

Cell migration was measured using the Transwell migration assay. 25 000 cells in serum‐free medium were added to the top chamber of the Transwell insert (Corning) placed in a 24‐well plate and allowed to migrate through the 8 μm polyethylene terephthalate (PET) membrane towards a chemoattractant (medium containing 10% FBS) in the bottom chamber for 20 to 24 h. Cells were fixed with 4% paraformaldehyde and stained with 0.1 μg·mL^−1^ DAPI. After removal of the cells that had not migrated, the migrated cells were imaged with the AMG EVOS FL microscope under a 4× lens and counted using imagej (National Institutes of Health, Bethesda, MA, USA). A total of three to four fields were photographed to cover the entire well. Cell migration was expressed as the number of migrated cells per field.

### Measurement of cell size

U87 and A172 cells were grown in 10 cm dishes to 60–70% confluence, collected by trypsinization and resuspended in 1× PBS (pH 7.4) to bring the final concentration to 1 × 10^6^ cells·mL^−1^. A total of 70 μL of cell suspension was loaded on a Moxi Z Cell Count Cassettes Type S (GeminiBio, LOT 2122), and cell volume was measured using a calibrated Moxi Z cell counter (Orflo, OS4.4, Ketchum, ID, USA). Data were analyzed using the moxichart software (Orflo, Verion 4.3.1065). The cell volume of the normal population of cells (identified by curve‐fitting function) was measured in triplicates for each biological replicate and the mean values were calculated.

### Sphere formation assay

Cells were cultured in sphere medium (DMEM/F12, 20 ng·mL^−1^ EGF, 20 ng·mL^−1^ FGF, and 1× B27 supplement) in 96‐well ultralow attachment plates at a density of 100 cells per well for U87 cells and 200 cells per well for U251 and A172 cells. The cells were allowed to grow into spheres for 10–14 days. The spheres were counted and imaged with an AMG EVOS FL microscope using a 4× lens. The size of the spheres was measured using imagej (RRID:SCR_003070).

### Mouse intracranial xenograft model

GBM cells at 80–90% confluency were trypsinized, washed twice with PBS, and resuspended in PBS at a concentration of 1 × 10^7^ (U87 cells) or 5 × 10^7^ cells (U251 and A172) per mL for intracranial injection into each male NSG (NOD‐SCID IL2R‐gamma null) mouse [39]. Briefly, mice subjected to 3% isoflurane in oxygen as anesthesia were positioned on a stereotactic platform for injection. A 1 cm sagittal incision over the parietal bone was made, and a burr hole 2 mm to the right of the midline and 1 mm posterior to the bregma (coronal suture) was drilled. 50 000 cells (U87 cells) or 250 000 cells (U251 and A172) in 5 μL of PBS were injected by syringe injector at a rate of 1 μL·min^−1^ into the skull at 3 mm depth followed by stapling of the scalp. After the entire volume was injected, the needle was left in the position for 1 min and then removed at a rate of 0.5 mm every 30 s. This allows the tissue to heal behind the needle without losing the cells through the injection site. U251 and U87 cells were stably transfected with luciferase. The growth and progression of the intracranial tumors were monitored weekly using IVIS bioluminescence imaging (BLI) as previously described [40]. The body weight of the mice was monitored weekly, and the mice were euthanized when weight loss reached 20% or when mice showed development of neurological symptoms. The time between cell injection and mouse euthanasia was recorded to generate survival curves of the animals in each experimental group. Mouse brains containing the tumors were collected, fixed, and sectioned. Human GBM cells in the brain sections were detected by immunohistochemistry (IHC) using an antibody that is specific for a human mitochondrial protein (clone 113‐1, Millipore Cat# MAB1273, RRID: AB_94052 at 1 : 1500 dilution).

The NSG mice were obtained from the breeding colony of the Dr. Postovit lab at the University of Alberta (AUP#1288). The breeders were purchased from the Charles River Laboratories (Wilmington, MA, USA). The animals were housed at a barrier facility of the vivarium at the CCI and handled by the experienced facility staff per approval of the animal protocol. The animal experiments were conducted with the approval of the CCI Animal Care and Use Committee, Edmonton, Canada (AC17232, approved on November 30, 2017) in accordance with guidelines from the Canadian Council for Animal Care. A172 cells were not transfected with luciferase. Mice injected with A172 cells were monitored by observing the body weight and development of neurological symptoms as described above. For the U251 tumor model, we first performed a pilot experiment using five mice for shControl and five mice for shNSUN5 group, and then predicted the minimum sample size for the second experiment based on the results of the experiment using the software medcalc (version 20.210, MedCalc Software Ltd, Ostend, Belgium). Accordingly, the minimum sample size for the second set of animal experiment was determined to be 5 and 7 for the U251 shControl and shNSUN5 group, respectively, based on the following assumptions: type I error 0.05; type II error 0.20. Difference of group means (35 days) as well as standard deviation of each group (shControl: 15 days; shNSUN5: 20 days) were estimated based on the results of the first animal experiment.

### Statistical analysis

Statistical significance between two groups was determined by Student's *t*‐test (two‐tailed distribution; two‐sample equal variance) and defined as *P* < 0.05. *, **, and *** indicate *P* < 0.05, *P* < 0.01, and *P* < 0.001, respectively. The statistical significance of survival difference in the animal experiments between the two groups was determined by the log‐rank test. Statistical analysis was performed using graphpad prism8 (RRID:SCR_002798). The correlation of NSUN5, STAT3, and NSUN2 mRNA and protein levels was analyzed using the Spearman's correlation coefficient.

## Results

### NSUN5 expression in GBM patients and cells

**Fig. 1:**
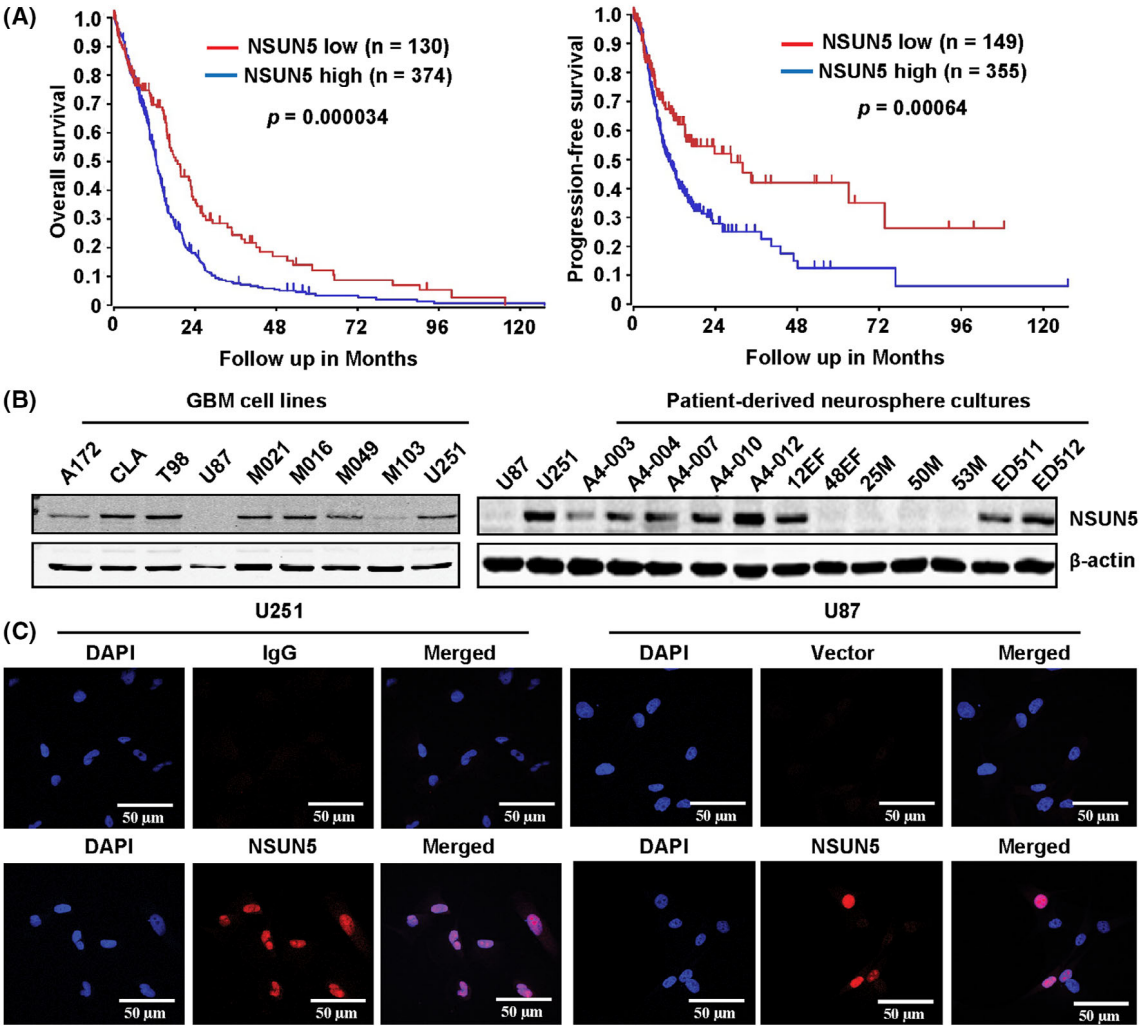
High NSUN5 mRNA levels are strongly associated with poor survival of GBM patients, and NSUN5 is expressed in GBM tissues and cells. (A) Kaplan–Meier curves were generated for GBM patients with low versus high NSUN5 mRNA levels by analyzing the GBM TCGA dataset from the R2 Genomics Analysis and Visualization Platform (https://hgserver1.amc.nl/cgi‐bin/r2/main.cgi). The cut‐off point for NSUN5 gene expression was determined by selecting the build‐in scan mode option in the platform, which provides the most significant gene expression cut‐off for survival analysis. High levels of *NSUN5* mRNA are strongly associated with shorter overall survival and progression‐free survival of GBM patients (*P* = 0.000034 and 0.00064). The statistical significance of survival difference between the two populations (NSUN5 low vs. high) was determined by the log‐rank test. (B) NSUN5 expression in nine GBM cell lines, and 12 primary patient‐derived neurosphere cultures was analyzed by Western blotting. β‐Actin was the loading control. Results are representative of two independent experiments. (C) Immunocytochemistry showed that both endogenous NSUN5 in U251 cells and ectopically expressed NSUN5 in U87 cells are localized in the nucleus. Scale bar = 50 μm. Results are representative of three independent experiments.

Analysis of a TCGA GBM dataset containing 153 patients showed that *NSUN5* mRNA levels, but not other members of the NSUN family, are strongly associated with the survival of GBM patients (Fig. S1A). Consistently, analysis of the GBM‐TCGA‐540 dataset (Affymetrix U133A mRNA expression profiling) using the R2 Genomics Analysis and Visualization Platform, confirmed that high *NSUN5* mRNA levels are strongly associated with poor overall and progression‐free survival in GBM patients (Fig. 1A). Analysis of this dataset showed that mRNA levels of *NSUN1* was also associated with shorter survival, but to a much less extent compared to *NSUN5*, whereas mRNA levels of *NSUN3* and *NSUN6* were associated with longer overall survival of GBM patients (Fig. S1B) Furthermore, analysis using the TCGA cohort (TCGA_GBM HG‐U133A) on Gliovis platform showed that *NSUN5* mRNA levels are higher in GBM tissues compared with nontumor tissues (Fig. S1C). The strong association between NSUN5 and poor survival in GBM patients using different datasets prompted us to ask whether elevated expression of NSUN5 plays a functional role in GBM. To address this question, we first examined the expression of NSUN5 in human GBM cell lines and GBM patient‐derived neurosphere cultures. Western blotting showed that NSUN5 is expressed in seven of nine GBM cell lines and eight of 12 patient‐derived neurosphere cultures (Fig. 1B). RT‐qPCR results showed that NSUN5 mRNA levels are consistent with the protein levels in the nine GBM cell lines tested (Fig. S1D). Immunocytochemistry showed that both endogenous NSUN5 in U251 cells and the ectopically expressed NSUN5 in U87 cells were localized in the nucleus (Fig. 1C), which is consistent with its function as RNA methyltransferase. IHC confirmed that NSUN5 is localized in the nucleus of cancer cells in human GBM tissues (Fig. S1E). Collectively, these results show that elevated NSUN5 expression is strongly associated with poor survival of GBM patients. Expression levels were therefore used to select appropriate cell models to investigate NSUN5 functions in GBM.

### NSUN5 methylates cytosine 3782 (C3782) of 28S rRNA in GBM cells

**Fig. 2:**
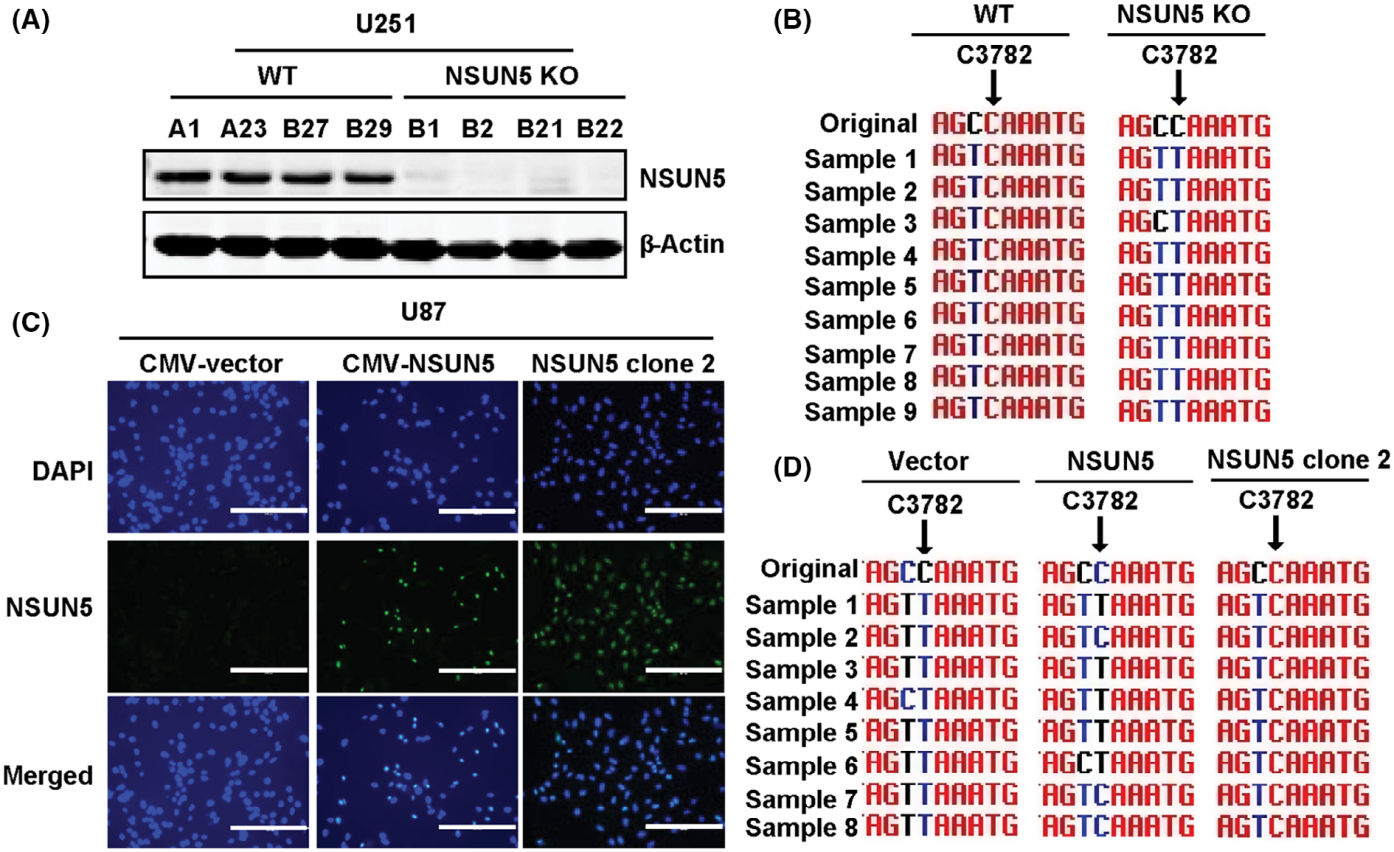
NSUN5 methylates C3782 of 28S rRNA in GBM cells. (A) Western blotting confirmed the expression NSUN5 in U251 WT and loss of NSUN5 in NSUN5 KO clones. Results are representative of three independent experiments. (B) Bisulfite sequencing showed that C3782 methylation is observed in U251 WT clones but lost in NSUN5 KO clones. (C) Immunocytochemistry was used to examine NSUN5 expression in U87 cells stably transfected with empty vector and NSUN5, as well as single cell NSUN5‐overexpressing clone (NSUN5 clone 2). DAPI was used to stain the nucleus. Scale bar = 200 μm. Results are representative of three independent experiments. (D) Bisulfite sequencing showed that C3782 is not methylated, partial methylated, and fully methylated in U87 vector, NSUN5 and NSUN5 clone 2 cells, respectively. KO, knockout; WT, wild‐type.

NSUN5 was predicted to methylate C3782 of 28S rRNA in mammals [15]. To determine whether this is the case in GBM cells, we used CRISPR/Cas9 to generate NSUN5 knockout (KO) and wild‐type (WT) clones in U251 cells, which was confirmed by genomic DNA sequencing (Fig. S2A) and Western blotting (Fig. 2A). RNA bisulfite sequencing showed that methylation of C3782 of 28S rRNA was observed in WT U251 cells, but not in NSUN5 KO U251 cells, indicating that NSUN5 KO leads to loss of C3782 methylation (Fig. 2B). We also confirmed that the ectopic expression of NSUN5 in U87 cells induced methylation of C3782 of 28S rRNA. Specifically, none of the eight samples selected from U87 cells transfected with the empty vector showed methylation of C3782 (Fig. 2C,D, left column). In contrast, three of the eight samples selected from U87 cells transfected with the NSUN5 expression construct, with ~ 40% of cells expressing NSUN5, displayed methylation of C782 (Fig. 2C,D, middle column). The eight samples selected from U87 clone 2 cells, with 100% of cells positive for NSUN5, displayed C3872 methylation (Fig. 2C,D, right column), indicating that C3782 methylation is dependent on the presence of NSUN5 in U87 cells. The Sanger sequencing chromatograms for Fig. 2B,D are shown in Figs S3 and S4, respectively. We also confirmed that NSUN1‐mediated C4447 methylation of 28S rRNA was not affected by NSUN5 KO in U251 or overexpression of NSUN5 in U87 cells (Fig. S5). Taken together, our results confirm that NSUN5 indeed methylates C3782 of 28S rRNA in GBM cells.

### NSUN5 increases global protein synthesis in GBM cells

**Fig. 3:**
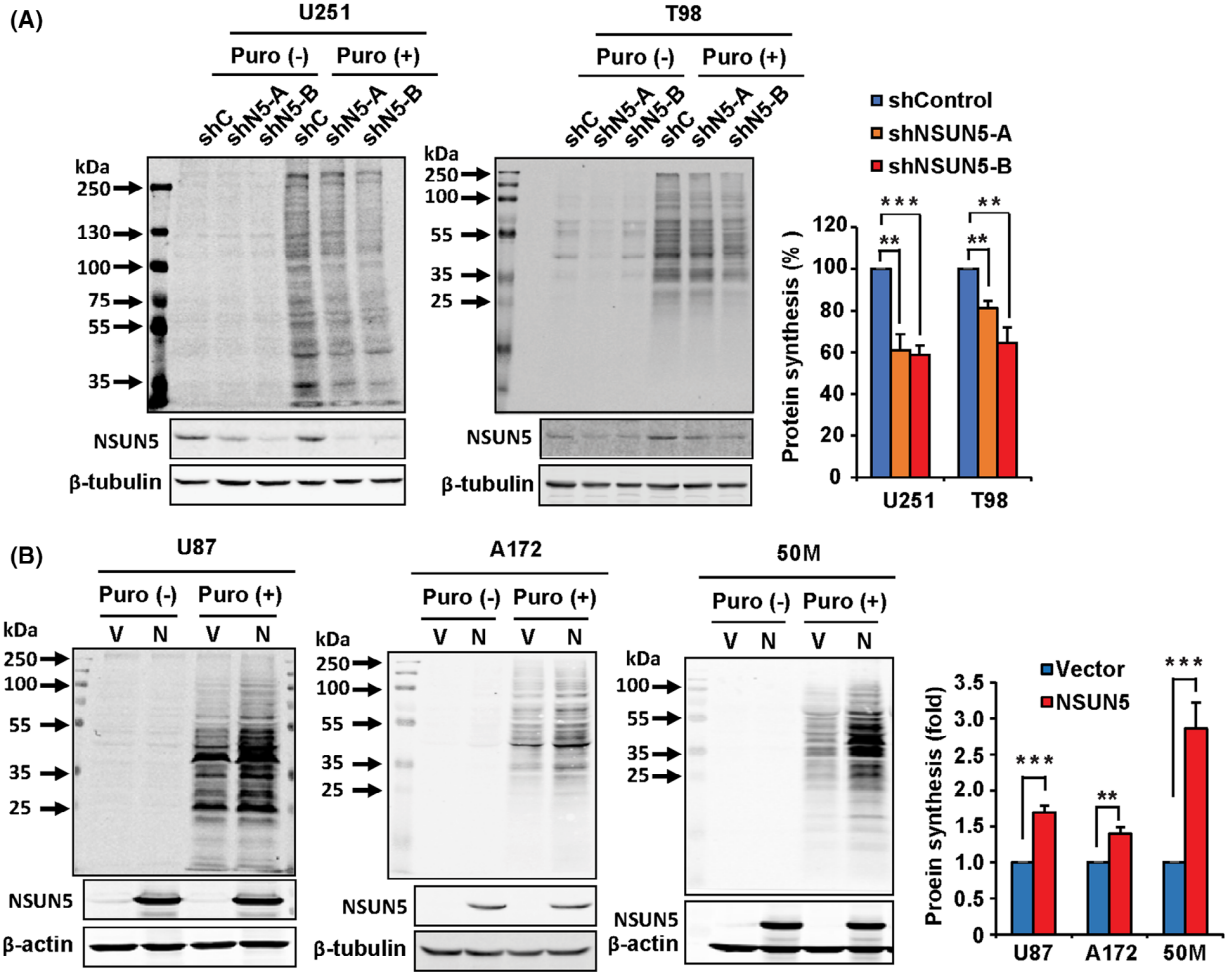
NSUN5 Increases global protein synthesis in GBM cells and primary patient‐derived neurospheres. The puromycin labeling assay was used to detect newly synthesized proteins in U251 and T98 cells stably transfected with shControl (shC), shNSUN5‐A (shN5‐A) and shNSUN5‐B (shN5‐B) (A) and in U87 and A172 cells, and 50M primary patient‐derived neurospheres stably transfected with vector (V) and NSUN5 (N) (B). Puromycin labeled proteins were detected by Western blotting using an antipuromycin antibody. NSUN5 expression and knockdown were confirmed using Western blotting. β‐Actin or β‐tubulin was used as loading controls. Puromycin incorporation was quantified by standardizing against the loading controls, and further expressed as percentage or fold against the shControl and vector cells, respectively. Data are mean ± SE of three (T98, U87), four (U251) and five (A172, 50M) independent experiments. Significantly different (Student's *t*‐test, **P < 0.01, ***P < 0.001). Puro (−): Vehicle control (ddH_2_O); Puro (+): Puromycin.

The role of NSUN5 in regulating protein synthesis was first reported in yeast [14]. To determine whether NSUN5 regulates global protein synthesis in GBM cells, we performed the puromycin‐labeling assay in which puromycin gets incorporated into nascent polypeptides and is detected by Western blotting. The puromycin‐labeling assays showed that stable knockdown of NSUN5 using two shRNA constructs (shNSUN5‐A and shNSUN5‐B) significantly decreased global protein synthesis in GBM cells (Fig. 3A). Specifically, quantification of the Western blotting results indicated that NSUN5 knockdown using shNSUN5‐A and ‐B resulted in a decrease in protein synthesis by 39% and 42%, respectively, in U251 cells, and by 19% and 35%, respectively, in T98 cells as compared to the shControl (Fig. 3A). Moreover, NSUN5 overexpression significantly increased global protein synthesis in GBM cells (Fig. 3B). Specifically, NSUN5 overexpression increased protein synthesis by 1.7‐, 1.3‐, and 2.8‐fold in U87, A172 and 50M cells, respectively, as compared to their respective empty‐vector controls (Fig. 3B). These results indicate that elevated NSUN5 expression leads to an increase in global protein synthesis in GBM cells.

### NSUN5 regulates the proteome in GBM cells

**Fig. 4:**
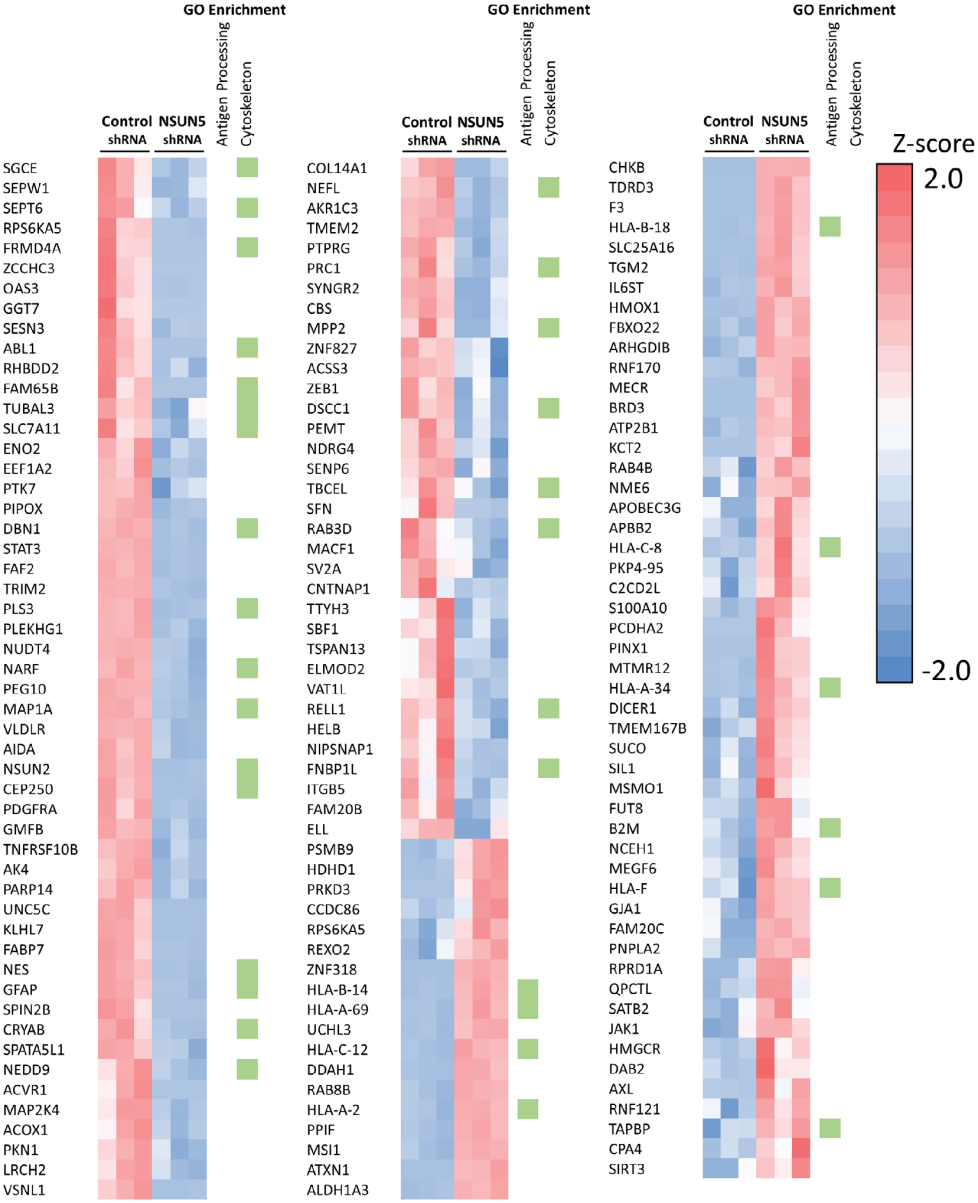
NSUN5 knockdown alters the proteome of glioblastoma cells. Heat map of the proteins identified to be significantly (*P* < 0.05) changed in expression by LFQ proteomic analysis upon shRNA knockdown of NSUN5 versus shRNA control in U251 cells. Cluster alignment of the data was performed with Clustergrammer. Columns represent quantified data from individual samples. Green boxes indicate the proteins that were identified in GO enrichment analysis.

Having shown that elevated NSUN5 increases protein synthesis, we next characterized the proteome regulated by NSUN5 in U251 and 50M cells using LC–MS/MS. Using this analysis, and normalizing for protein concentration, we detected relatively few differentially regulated proteins, indicating that NSUN5 likely regulates global translation. There were, however, some differentially expressed proteins: NSUN5 knockdown led to statistically significant downregulation of 87 proteins and upregulation of 69 proteins in U251 cells (Fig. 4). GO enrichment analysis showed that the downregulated proteins in shNSUN5‐B cells were enriched for cytoskeletal association and the upregulated proteins in shNSUN5‐B were enriched for antigen processing (Fig. 4). NSUN5 overexpression significantly increased the expression of 47 proteins and downregulated the expression of 25 proteins in 50M cells (Fig. S6). GO enrichment analysis showed that the altered proteins in 50M NSUN5 cells were associated with alternative splicing functions (Fig. S6). The proteomic data are also presented as volcano plots in Fig. S7. From the significantly decreased proteins in response to NSUN5 knockdown in U251 cells, we selected STAT3 and NSUN2 for validation by Western blotting and RT‐qPCR, because the protumorigenic functions of STAT3 in GBM has been well‐documented [41] and NSUN2 (another member of the NSUN family) has been shown to increase migration of U87 cells [42]. The LC–MS/MS data showed that NSUN5, STAT3 and NSUN2 were decreased in U251 shNSUN5‐B cells compared with shControl cells by 72%, 72% and 62%, respectively (Fig. S8A). Western blotting confirmed that NSUN5 knockdown decreased NSUN5, STAT3, and NSUN2 protein levels in U251 and T98 cells (Fig. S8B,D). Importantly, the mRNA levels of NSUN5, STAT3, and NSUN2 were also decreased in NSUN5 knockdown U251 and T98 cells (Fig. S8C,E) and the decrease in mRNA and protein levels was significantly correlated (Fig. S9), suggesting that the decrease in STAT3 and NSUN2 proteins in NSUN5 knockdown cells may be due to factors that alter the transcriptome. Furthermore, Western blotting confirmed that knockdown of NSUN5 led to decreased protein levels of some key factors in GBM, such as Nestin, pSTAT3, PDGFRA, and FABP7, in U251 cells (Fig. S10).

### NSUN5 knockdown mitigates the tumorigenic phenotypes of GBM cells *in vitro*

**Fig. 5:**
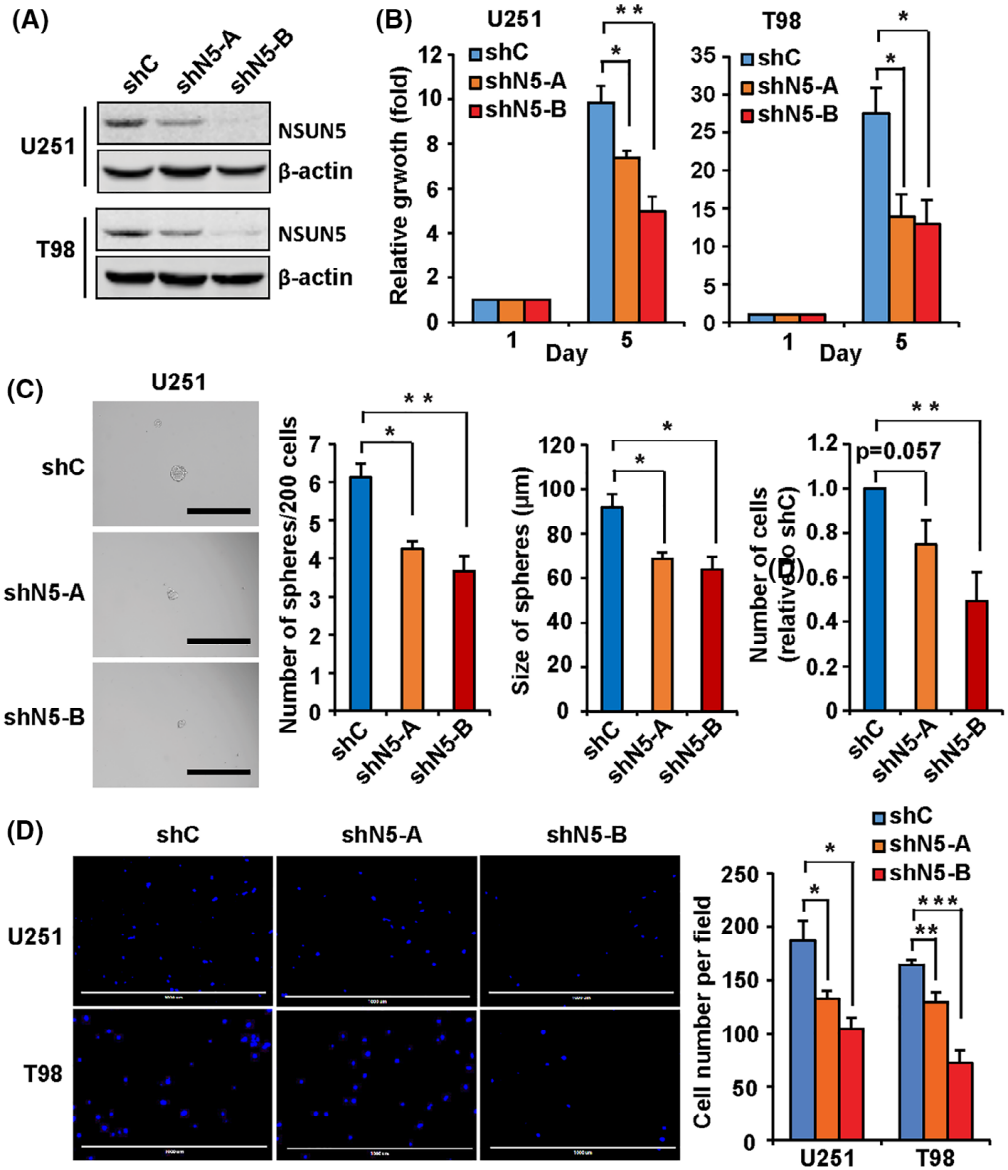
NSUN5 Knockdown decreases the growth, sphere formation, and migration in GBM cells. (A) Western blotting confirmed the knockdown of NSUN5 in U251 and T98 cells. β‐Actin was used as the loading control. (B) Neutral red uptake assay showed that NSUN5 knockdown via shN5‐A and ‐B significantly decreased the growth, as compared to shC in U251 and T98 cells at day 5. Cell growth was quantified by standardizing against day 1 and shown as the fold change. (C) Sphere‐formation assay showed that NSUN5 knockdown decreased the number and size of spheres, as well as the number of viable U251 cells in the spheres (determined by the neutral red uptake assay and normalized against shC). Images of spheres were taken using the EVOS inverted microscope under 10× magnification. Scale bar = 400 μm. The size of spheres was measured using imagej. (D) Transwell migration assay showed that NSUN5 knockdown decreased the number of migratory cells for U251 and T98, as compared to shC. Cells were stained with DAPI. Images were taken using the EVOS inverted microscope under 4× magnification. Scale bar = 1000 μm. The number of migrated cells were counted using ImageJ. Cell migration was expressed as the average number of cells per field. Data are mean ± SE of four experiments. Significantly different (Student's *t*‐test, *P < 0.05, **P < 0.01, ***P < 0.001). shC, shControl; shN5‐A, shNSUN5‐A; shN5‐B, shNSUN5‐B.

Because protein synthesis is commonly deregulated in cancer to support their growth, we examined the functional impact of the elevated NSUN5 expression in GBM cells. NSUN5 knockdown in U251 and T98 cells using shNSUN5‐A and ‐B (Fig. 5A) reduced the number of viable cells by 25% and 50%, respectively, in U251 cells and by 49% and 53%, respectively, in T98 cells when compared to their respective controls (Fig. 5B). GBM cancer stem cells (GSCs) contribute to GBM initiation, progression, resistance to therapies, and recurrence [43]. To determine whether NSUN5 promotes the self‐renewal and proliferation of GSCs, we performed sphere‐formation assays in ultra‐low attachment 96‐well plates in sphere culture conditions. NSUN5 knockdown using shNSUN5‐A and ‐B decreased the number of U251 spheres by 27% and 38%, respectively, and the size of U251 spheres by 25% and 30%, respectively, as compared to the shRNA control (Fig. 5C). We also used the neutral red uptake assay to quantify the number of viable cells in the spheres, which showed that NSUN5 knockdown using shNSUN5‐A and ‐B decreased the number of U251 cells in the spheres by 25% and 51%, respectively, as compared to the shRNA control (Fig. 5C). T98 cells did not form spheres even at 1000 cells per well under the same culture conditions as for U251 cells. GBM is characterized by infiltrative growth in the brain and cell migration is a critical process in tumor progression of GBM [44]. Transwell migration assays showed that NSUN5 knockdown using shNSUN5‐A and ‐B decreased migration by 35% and 34%, respectively, in U251 cells and by 29% and 41%, respectively, in T98 cells as compared to the shRNA control (Fig. 5D). TMZ is the first‐line chemotherapeutic agent for GBM patients; however, the development of resistance to TMZ via multiple mechanisms limits its efficacy [45]. TMZ causes cell death by inducing methylation at the O^6^ position of guanine, which can be removed by MGMT. U251 cells lack the expression of MGMT, whereas T98 cells express a high level of MGMT, which render these cells sensitive and resistant to TMZ treatment, respectively [45, 46]. We treated the TMZ‐sensitive U251 cells and TMZ‐resistant T98 cells with increasing concentrations of TMZ and showed that NSUN5 knockdown rendered U251 and T98 cells more sensitive to TMZ treatment as determined by the neutral red uptake and clonogenic assays (Fig. S11). Taken together, these results indicate that NSUN5 contributes to the tumorigenic phenotypes in GBM *in vitro*.

### The effect of NSUN5 overexpression on the behavior of GBM cell lines

**Fig. 6:**
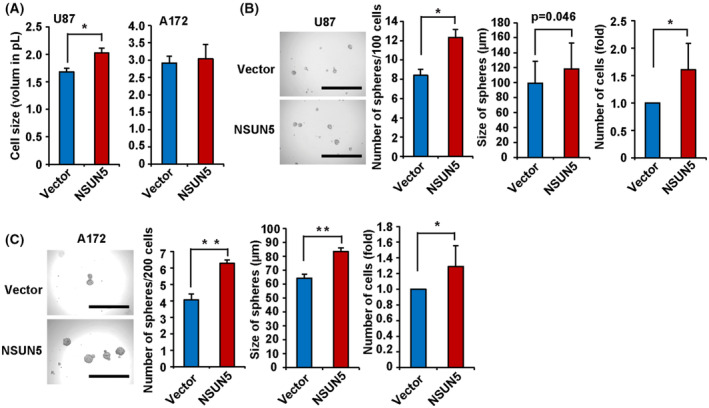
Effect of ectopic expression of NSUN5 on the GBM phenotypes. (A) The ectopic expression of NSUN5 increased the size of U87 cells, but not that of A172 cells, as compared to their respective vector cells. (B, C) The sphere‐formation assay showed that the ectopic expression of NSUN5 increased the number and size of spheres, and the number of viable cells (determined by the neutral red uptake assay) for U87 and A172 cells. The number of viable cells were quantified by standardizing against the vector control. Images of U87 and A172 spheres were taken using the EVOS inverted microscope under 4×, respectively. Scale bar = 1000 μm. The size of spheres was measured using imagej. Data are mean ± SE of three (A, C) and four (B) independent experiments. Significantly different (Student's *t*‐test, *P < 0.05, **P < 0.01).

To further explore the role of NSUN5 in regulating GBM phenotypes, we ectopically expressed NSUN5 in U87 and A172 cells (Fig. 3B). Immunocytochemistry confirmed that more than 90% of the cells were stably transfected with NSUN5 in both U87 and A172 cells (Fig. S12). The neutral red uptake assays showed that NSUN5 overexpression in U87 and A172 cells did not change the number of viable cells in adherent cultures (Fig. S13). Cell size analysis showed that NSUN5 overexpression increased the size of U87 cells by 1.2‐fold but did not change the size of A172 cells in adherent cultures (Fig. 6A). Interestingly, NSUN5 overexpression increased the size and number of U87 and A172 spheres when cultured in the sphere‐forming conditions in ultra‐low attachment plates (Fig. 6B,C). Specifically, NSUN5 overexpression increased the number of spheres by 1.5‐fold in U87 cells and 1.6‐fold in A172 cells and increased the size of the spheres by 1.2‐fold in U87 cells and 1.3‐fold in A172 cells (Fig. 6B,C). Furthermore, the neutral red uptake assays showed that NSUN5 overexpression increased the number of viable cells in the spheres by 1.6‐fold in U87 cells and by 1.8‐fold in A172 cells (Fig. 6B,C).

### NSUN5 enhances the growth of primary patient‐derived neurospheres

**Fig. 7:**
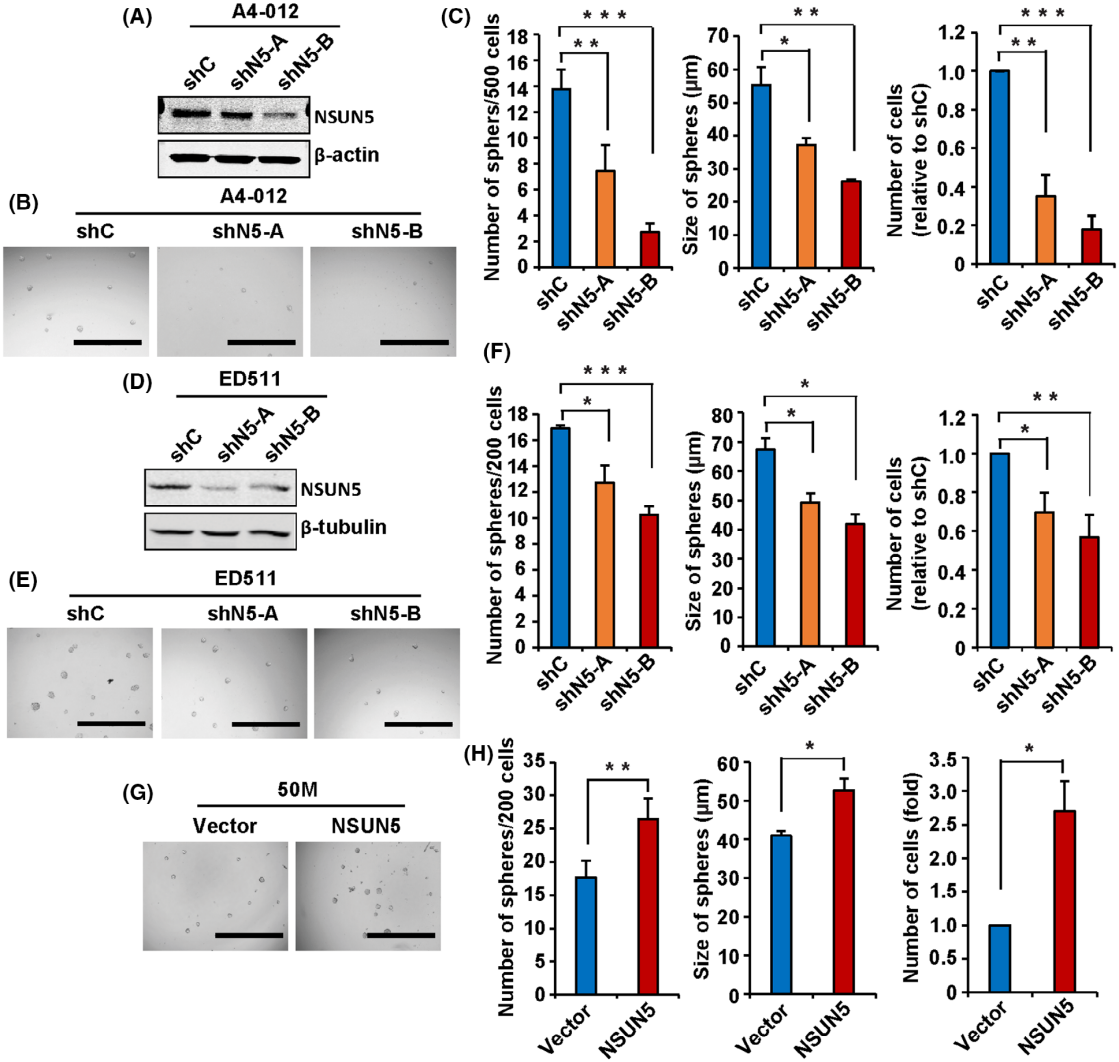
NSUN5 Increases the growth of primary patient‐derived neurospheres. (A, D) Western blotting confirmed knockdown of NSUN5 in A4‐012 and ED511 neurospheres. β‐Actin and β‐tubulin were used as the loading control. NSUN5 knockdown decreased the number and size, as well as the number of viable cells in A4‐012 neurospheres (B, C) and in ED511 neurospheres (E, F). (G, H) NSUN5 overexpression increased the number and size, as well as the number of viable cells in 50M neurospheres. Images were taken using the EVOS inverted microscope under 4× magnification. Scale bar = 1000 μm. The size of spheres was measured using imagej. Data are mean ± SE of three (A4‐012, ED511) and four (50M) independent experiments. Significantly different (Student's *t*‐test, *P < 0.05, **P < 0.01, ***P < 0.001). shC, shControl; shN5‐A, shNSUN5‐A; shN5‐B, shNSUN5‐B.

Increasing evidence suggests that patient‐derived neurospheres are a more clinically relevant model for GBM as compared to established cell lines, as they are more likely to maintain the stemness of GBM stem cells [47, 48]. NSUN5 knockdown using shNSUN5‐A and ‐B decreased the number of A4‐012 neurospheres by 46% and 80%, respectively, and the size of the spheres by 33% and 52%, respectively, as compared to shControl (Fig. 7A–C). Similarly, NSUN5 knockdown by shNSUN5‐A and ‐B decreased the number of ED511 neurospheres by 25% and 39%, respectively, and the size of the spheres by 27% and 38%, respectively, as compared to shControl (Fig. 7D–F). The neutral red uptake assays showed that NSUN5 knockdown by shNSUN5‐A and ‐B decreased the number of viable cells in A4‐012 spheres by 65% and 82% and in ED511 spheres by 42% and 53%, respectively (Fig. 7C,F). Furthermore, we found that NSUN5 overexpression increased the size and number of 50M neurospheres by 1.3‐ and 1.5‐fold, respectively, as compared to the empty‐vector control, and the number of viable cells in 50M spheres by 2.7‐fold as determined by the neutral red uptake assay (Fig. 7G,H). Taken together, our results indicate that NSUN5 enhances the self‐renewal and proliferation of patient‐derived GBM neurosphere cultures.

### Mice bearing intracranial U251 shNSUN5 tumors survive longer than mice bearing U251 shControl tumors

**Fig. 8:**
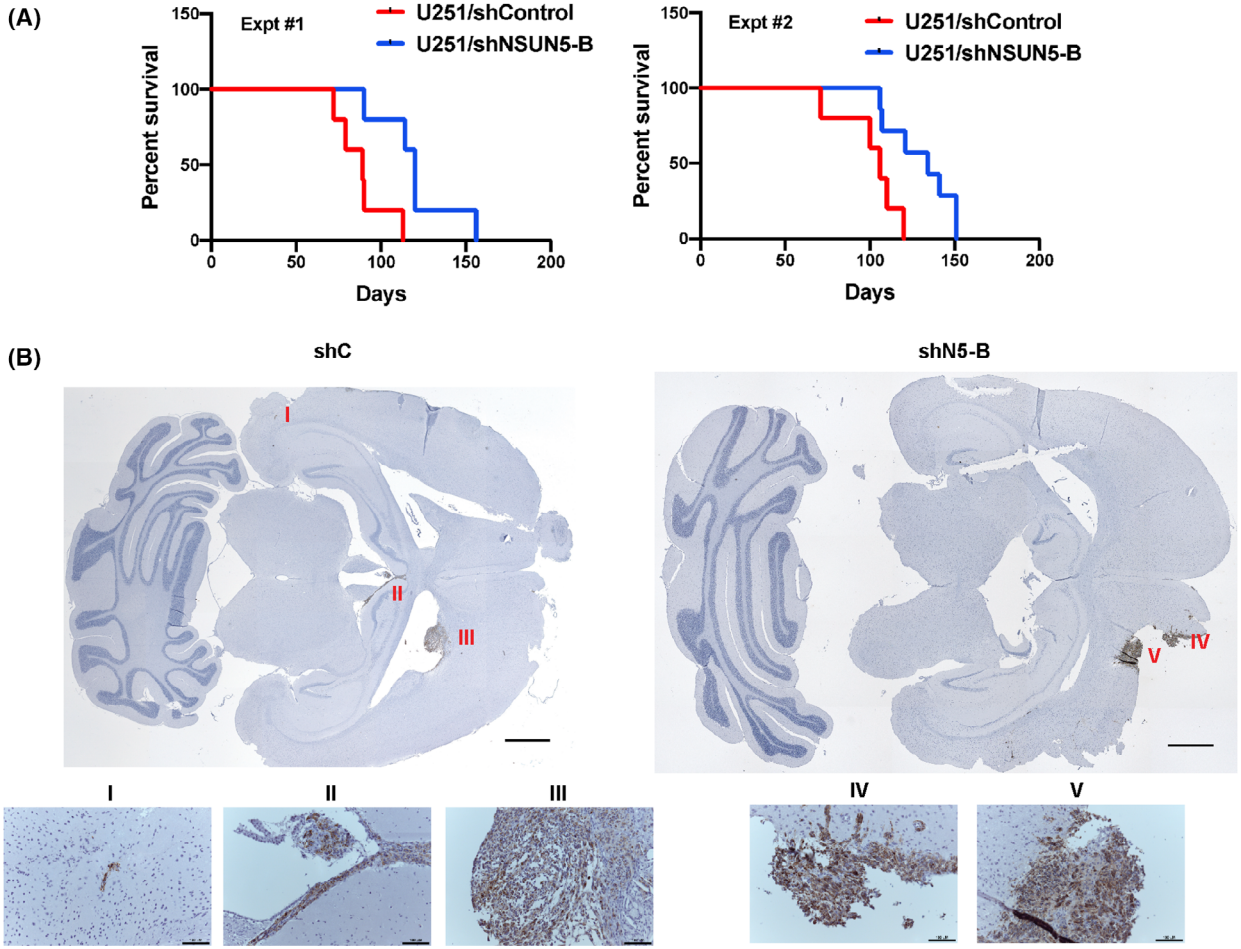
NSUN5 knockdown prolongs the survival of mice bearing U251 intracranial tumors, likely through decreased migration. (A) Kaplan–Meier survival curves of NSG mice bearing the intracranial U251 shControl (shC) and shNSUN5‐B (shN5‐B) tumors in two independent experiments. Mice bearing the U251 shNSUN5‐B tumors had a longer survival time compared to mice bearing the U251 shControl tumors. The statistical significance of survival difference between the two groups was determined by the log‐rank test: *P* = 0.0088 for the first experiment and *P* = 0.013 for the second experiment. (B) IHC of the U251 xenografted tumors using an antibody that specifically detects a human mitochondria protein (stained in brown). Leptomeningeal metastases were observed for shC cells, in which the neoplastic cells were detected in (I) subependymal layer of the left lateral ventricle; (II) the ependymal layer of the third ventricle; and (III) the right frontal horn of the lateral ventricle. The shN5‐B cells were more localized, in which the neoplastic cells were detected only in the parenchyma of the frontal lobe (IV and V). Scale bar = 1 mm for the top images. Scale bar = 100 μm for the bottom images.

To assess the function of NSUN5 *in vivo*, we used an orthotopic xenograft model, in which GBM cells were implanted into NSG (NOD scid gamma) mice via intracranial injection. Results of two independent experiments showed that mice bearing the U251 shNSUN5‐B tumors had a longer survival time compared to mice bearing the U251 shControl tumors; the median survival was 120 days (*n* = 5) vs 89 days (*n* = 5, *P* = 0.0088) in the first experiment and 134 days (*n* = 5) vs 106 days (*n* = 7, *P* = 0.013) in the second experiment (Fig. 8A). IHC using an antibody specific for human mitochondria showed that U251 shNSUN5‐B tumors were localized near the injection site, whereas the U251 shControl tumors had intra‐organ invasion, forming distal tumors in both the ependymal and leptomeningeal layers of the brain (Fig. 8B and Fig. S14). IHC staining for NSUN5 confirmed NSUN5 knockdown in U251/shNSUN5 tumors (Fig. S15). *In vivo* BLI showed that there was no difference in bioluminescent signal intensities between the U251 shNSUN5‐B and shControl tumors (Fig. 16A,B). Hence, the IHC and BLI results suggest that the difference in survival time between mice bearing U251 shControl and shNSUN5‐B tumors is not caused by the tumor mass, but likely due to the more migratory and invasive phenotype of U251 shControl cells, which is supported by our *in vitro* migration assays where knockdown of NSUN5 decreased migration of U251 cells (Fig. 5D). Interestingly, *in vivo* BLI showed that eight of 10 mice bearing the U251 shControl tumors had spinal metastasis as compared to only two of 12 mice bearing the U251 shNSUN5‐B tumors (Fig. S16C), which is significantly different (Fisher test, *P* = 0.0083), suggesting that U251 shControl cells are more migratory *in vivo*. However, NSUN5 overexpression did not impact the survival of mice bearing intracranial U87 and A172 tumors (Fig. S17). Taken together, our results suggest that NSUN5 knockdown decreases U251 cell migration in the intracranial tumor model; however, overexpression of NSUN5 does not affect the survival of mice bearing the U87 and A172 tumors.

## Discussion

In this study we demonstrated that NSUN5 plays a protumorigenic role in GBM, concomitant with enhancing protein synthesis. This finding is supported by other studies showing that other NSUN proteins, in addition to well‐studied NSUN2, are implicated in cancer [20, 21, 22, 49, 50, 51, 52]. In cancer cells, mRNA translation is often dysregulated, leading to increased overall protein synthesis and/or selective expression of pro‐tumorigenic mRNAs to promote proliferation and survival of cancer cells, as well as the adaptation to the tumor microenvironment and therapy‐induced stress [53, 54]. Most studies have focused on the regulation of translation initiation as a rate limiting step; however, our results suggest that NSUN5 may regulate protein synthesis by broadly affecting protein synthesis. This led to a variety of phenotypes, including increased proliferation, enhanced cell size, and increased migratory potential. Collectively, these contributed to an enhancement of pro‐tumorigenic phenotypes such as sphere formation, migration and invasion in a mouse model of GBM. Our results also support a role for NSUN5 in promoting TMZ resistance in GBM cells. The mechanisms of TMZ resistance are multifactorial, including *MGMT* promoter methylation status [45]. A recent study showed that loss of NSUN6 (another NSUN family member) renders GBM cells more resistant to TMZ as a consequence of cytosine methylations introduced in RNAs that are involved in transcriptional and translational initiation, regardless of MGMT methylation status [49]. Our finding that knockdown of NSUN5 sensitizes both U251 and T98 cells, two cell lines with different MGMT expression and sensitivity to TMZ [46] suggests that NSUN5‐mediated TMZ resistance is independent of MGMT expression. GBM cells maintained under neurosphere conditions more closely represent the genotype and phenotype of primary tumors, especially the stemness of cancer stem cells, compared to GBM cell lines [47, 48]. In accordance with our observations in the GBM cell lines, NSUN5 knockdown decreased, whereas overexpression of NSUN5 increased, protein synthesis and the number and size of the GBM neurospheres, further supporting a protumorigenic role for NSUN5 in GBM.

Our *in vivo* results show that mice bearing the intracranial NSUN5 knockdown U251 tumors survived longer compared with mice bearing U251 control tumors. However, there was no difference in tumor bioluminescence intensities between the control and NSUN5 knockdown tumors, indicating that the difference in survival cannot be attributed to the difference in tumor mass or the number of the tumor cells between the two groups. IHC showed that NSUN5 knockdown U251 tumors tended to grow close to the injection site, whereas the control tumors migrated to the subarachnoid space at multiple sites in the brain through the cerebrospinal fluid circulation and the ventricular system, which is consistent with decreased migration in NSUN5 knockdown U251 cells *in vitro*. Another interesting observation is that 8 of 10 mice bearing control U251 tumors, but only 2 out of 12 mice bearing NSUN5 knockdown U251 tumors, displayed spinal metastasis. However, with the current injection model (not image‐guided injection) and the tumor IHC data, we cannot exclude the possibility that the tumors observed at multiple sites and the spinal metastases could be formed by the cells that were unintentionally spilled out of injection sites. Nevertheless, the finding that U251 shControl cells formed tumors at multiple locations and displayed more leptomeningeal and spinal metastases than U251 shNSUN5 cells indicates that knockdown of NSUN5 decreased migration of U251 cells *in vivo*. Clinical studies indicate that leptomeningeal and spinal metastases are observed in 4–25% of GBM patients [55, 56]. The tumor cells in the brain can migrate from the superior temporal and central sulcus, the transverse and cortical fissures, and the lateral ventricle to the CSF and the spine to form metastases [56]. Clinical studies show that spinal metastasis is not associated with the overall survival of GBM patients, which is likely due to the rapid progression of GBM in the brain and the poor overall survival of GBM patients [56, 57]. Similarly, in our study, spinal metastasis of the U251 tumors was not associated with the survival of the mice, suggesting that it is not the cause of death; rather it is a manifestation of control U251 cells being more migratory in the brain compared to NSUN5 knockdown U251 cells.

Our results show that, in contrast to the NSUN5 dependency observed in the cell lines that express endogenous NSUN5, introduction of NSUN5 into cell lines that have evolved and adapted to survive without NSUN5 may confer certain advantages to the cells, but not in all the biological processes we investigated in this study. In this regard, we found that NSUN5 expression resulted in increased U87 cell size consistent with the report in HeLa cells [20], and increased sphere formation in both U87 and A172 cells. However, it does not change their proliferation in adherent culture conditions, nor does it have an impact on the survival of mice bearing intracranial U87 or A172 tumors. These results indicate a context‐dependent effect of overexpressed NSUN5 in GBM.

Thus far, only three studies on NSUN5 in cancer have been reported. These studies showed that NSUN5 promotes cell proliferation in colorectal cancer [22], and proliferation and cell size in HeLa cells [20], but inhibits cell proliferation and tumor progression in GBM [21]. Our results suggest that NSUN5 plays a protumorigenic role in GBM cells, which is in line with the strong association between the elevated NSUN5 expression and shorter overall and disease‐free survival of GBM patients [21, 58] and in accordance with the studies in colorectal cancer and cervical cancer [20, 22]. Janin et al. reported that NSUN5 methylates C3782 of 28S rRNA and promotes protein synthesis in GBM cells, which is consistent with our findings in this study. Interestingly, however, contradictory to our findings, they showed that NSUN5 plays a tumor suppressing role in GBM: NSUN5 overexpression decreases, whereas NSUN5 knockdown increases, cell proliferation *in vitro* and tumor formation and progression of GBM cells *in vivo* [21]. In both studies, A172 cells were used for overexpression experiments. We found that overexpression of NSUN5 in A172 cells increased sphere formation but did not affect cell proliferation in adherent culture *in vitro*, nor did it affect the survival of mice bearing A172 intracranial tumors, whereas Janin et al. showed that overexpression of NSUN5 in A712 cells repressed cell proliferation *in vitro* and tumor size *in vivo* [21]. Hence, the discrepancy between the two studies cannot be explained by the use of different cell lines. However, the protumorigenic functions of NSUN5 we observed in our study is in accordance with the strong association between NSUN5 expression and shorter survival of GBM patients, as well as with the role of NSUN5 in promoting protein synthesis in GBM cells.

There are some limitations to this study. First, we measured cell growth using the neutral red uptake assay, which does not distinguish the effect of NSUN5 on cell proliferation and survival. Although this does not change our conclusions, further analysis, such as cell cycle analysis, may help elucidate the molecular mechanisms underlying the protumorigenic functions of NSUN5 in GBM. Second, we have determined that NSUN5 regulates the expression of some key factors in U251 cells. It would be important to further investigate this regulation in primary neurosphere cultures and to determine whether these factors mediate the protumorigenic functions of NSUN5 in GBM tumors. Third, we showed that NSUN5 regulates protein synthesis in GBM, however, the underlying mechanisms remain to be elucidated. For example, it remains to be determined whether NSUN5‐mediated methylation of C3872 of 28S rRNA affects the composition of ribosomal proteins in the ribosome, thereby changing the structure and functions of the ribosome. Finally, due to limited availability of proteomic datasets suitable for survival analysis, it remains to be determined whether the NSUN5 protein levels are associated with GBM patient survival. Using integrated multi‐omics analysis, Wang et al. recently identified key players in the oncogenic pathways involved in different subtypes of GBM and potential targets for GBM treatment [24]. Our analysis using the proteomic dataset reported by Wang et al. showed that NSUN5 protein levels are not associated with the survival of GBM patients in this cohort (*n* = 99; Fig. S18). As GBM is a highly heterogeneous tumor, it will be important to carry out additional analysis using GBM protein datasets as they become available in order to further investigate the correlation between NSUN5 at the protein level and GBM patient survival.

## Conclusions

Our study shows that NSUN5 is an RNA cytosine methyltransferase that mediates cytosine 3782 methylation of 28S rRNA and promotes protein synthesis and tumorigenic properties in GBM, providing additional support for the emerging role of RNA 5‐methylcytosine in cancer. Further studies are warranted to determine the therapeutic application of targeting NSUN5 in GBM.

## Conflict of interest

The authors declare no conflict of interest.

## Author contributions

JZ and YSK were involved in formal analysis, investigation, methodology, validation, and writing—original draft. KMV was involved in formal analysis and conceptualization. DDC, ABB, DG, and DQ were involved in formal analysis, investigation, and methodology. R‐ZL and RF were involved in formal analysis and writing—review and editing. SDF, GL, and AMG were involved in methodology. XH, ZX, RZY, LZ, and ET were involved in investigation. DDE was involved in funding acquisition, conceptualization, and writing—review and editing. RG was involved in conceptualization, funding acquisition, supervision, and writing—review and editing. L‐MP. Postovitwas involved in conceptualization, funding acquisition, project administration, supervision, and writing—review and editing. YXF was involved in conceptualization, funding acquisition, project administration, supervision, and writing—review and editing.

### Peer review

The peer review history for this article is available at https://www.webofscience.com/api/gateway/wos/peer‐review/10.1002/1878‐0261.13434.

## Supporting information


**Fig. S1.** Expression of NSUN5 in GBM patients.
**Fig. S2.** Genomic DNA sequencing of U251 NSUN5 KO clones.
**Fig. S3.** Sanger Sequencing chromatograms of the bisulfite sequencing results of U251 WT and KO cells.
**Fig. S4.** Sanger Sequencing chromatograms of the bisulfite sequencing results of U87 cells.
**Fig. S5.** NSUN1‐mediated C4447 methylation of 28S rRNA was not affected by NSUN5 KO in U251 cells or overexpression in U87 cells.
**Fig. S6.** Heatmap of the proteins altered by NSUN5 overexpression in 50M cells.
**Fig. S7.** Volcano plots of the proteins altered by NSUN5 knockdown in U251 and NSUN5 overexpression in 50M cells.
**Fig. S8.** NSUN5 knockdown leads to decreased expression of STAT3 and NSUN2.
**Fig. S9.** Levels of mRNA and protein are significantly correlated for NSUN5, STAT3 and NSUN2.
**Fig. S10.** NSUN5 knockdown leads to decreased protein level of some key factors in GBM.
**Fig. S11.** Knockdown of NSUN5 sensitizes GBM cells to temozolomide.
**Fig. S12.** Immunocytochemistry of the overexpressed NSUN5 in U87 and A172 cells.
**Fig. S13.** Overexpression of NSUN5 does not affect cell growth in U87 and A172 cells cultured in adherent conditions.
**Fig. S14.** U251 tumors in the brain of the mice.
**Fig. S15.** Expression of NSUN5 in U251/shControl and U251/shNSUN5 tumors.
**Fig. S16.** Analysis of bioluminescence imaging of U251 tumors.
**Fig. S17.** Overexpression of NSUN5 did not change the survival of mice bearing U87 and A172 tumors.
**Fig. S18.** Association between NSUN5 protein level and survival of GBM patients.

**Table S1.** Sequencing of gRNAs, shRNAs, and PCR primers.

**Table S2.** Instrument parameters for data acquisition.

**Table S3.** Raw data of proteomic analysis in U251 cells.

**Table S4.** Raw data of proteomic analysis in 50M cells.

## Data Availability

The raw data of the proteomic analysis were provided in Tables S3 and S4.
