# Supplementary material for: RNA cytosine methyltransferase NSUN5 promotes protein synthesis and tumorigenic phenotypes in glioblastoma
Source: Mol Oncol. 2023 Apr 22;17(9):1763–83. doi: 10.1002/1878-0261.13434 (PMC10483612; doi:10.1002/1878-0261.13434)
Supplement: Supplementary file 1 — Fig. S1. Expression of NSUN5 in GBM patients. Fig. S2. Genomic DNA sequencing of U251 NSUN5 KO clones. Fig. S3. Sanger Sequencing chromatograms of the bisulfite sequencing results of U251 WT and KO cells. Fig. S4. Sanger Sequencing chromatograms of the bisulfite sequencing results of U87 cells. Fig. S5. NSUN1‐mediated C4447 methylation of 28S rRNA was not affected by NSUN5 KO in U251 cells or overexpression in U87 cells. Fig. S6. Heatmap of the proteins altered by NSUN5 overexpression in 50M cells. Fig. S7. Volcano plots of the proteins altered by NSUN5 knockdown in U251 and NSUN5 overexpression in 50M cells. Fig. S8. NSUN5 knockdown leads to decreased expression of STAT3 and NSUN2. Fig. S9. Levels of mRNA and protein are significantly correlated for NSUN5, STAT3 and NSUN2. Fig. S10. NSUN5 knockdown leads to decreased protein level of some key factors in GBM. Fig. S11. Knockdown of NSUN5 sensitizes GBM cells to temozolomide. Fig. S12. Immunocytochemistry of the overexpressed NSUN5 in U87 and A172 cells. Fig. S13. Overexpression of NSUN5 does not affect cell growth in U87 and A172 cells cultured in adherent conditions. Fig. S14. U251 tumors in the brain of the mice. Fig. S15. Expression of NSUN5 in U251/shControl and U251/shNSUN5 tumors. Fig. S16. Analysis of bioluminescence imaging of U251 tumors. Fig. S17. Overexpression of NSUN5 did not change the survival of mice bearing U87 and A172 tumors. Fig. S18. Association between NSUN5 protein level and survival of GBM patients. [file MOL2-17-1763-s004.pdf]

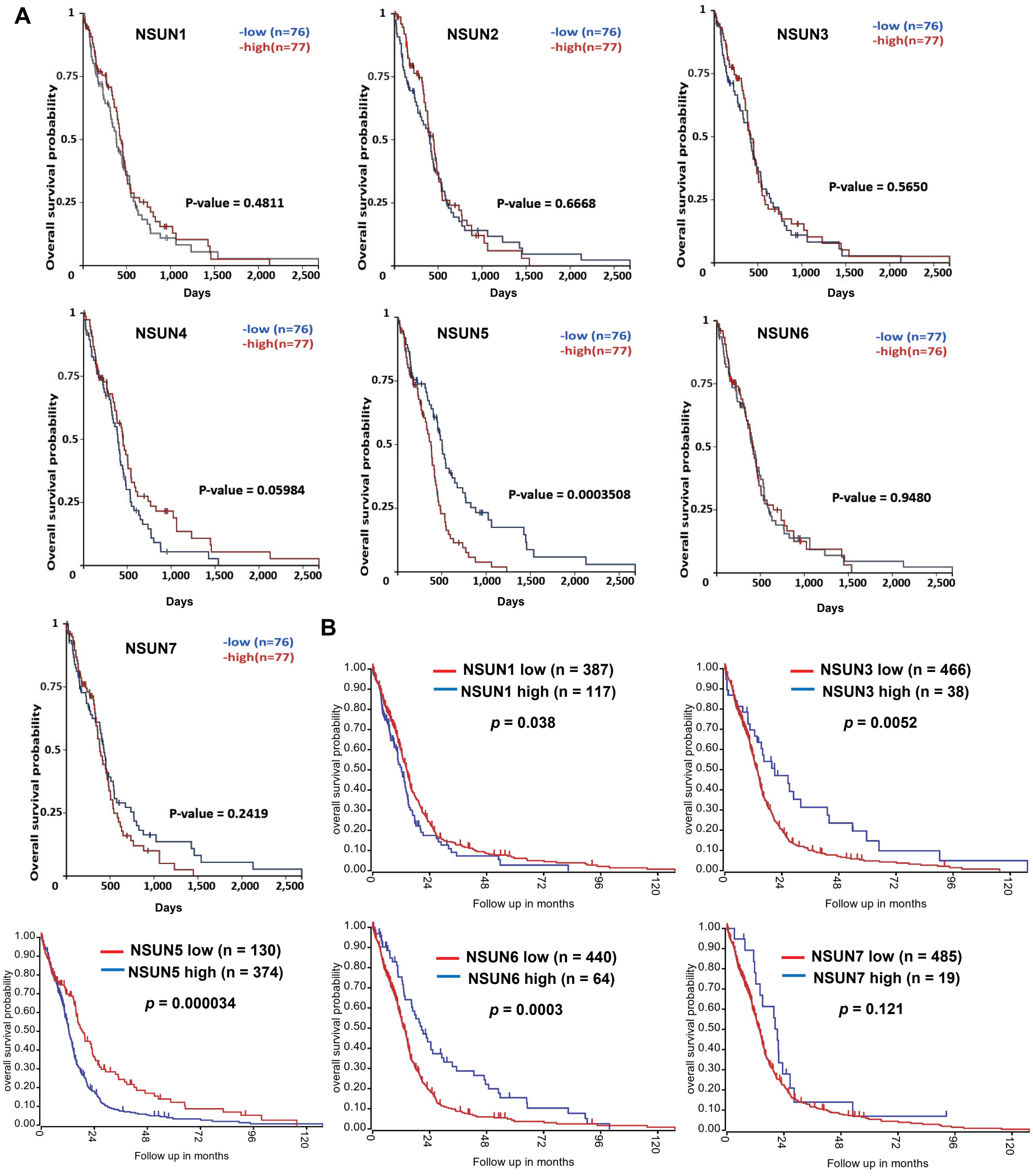

Supplementary Figure 1. Expression of NSUN5 in GBM patients. (A) The TCGA GBM dataset containing 153 patients was analyzed to determine the correlation between the mRNA expression of NSUN1-7 with the survival of GBM patients using the UCSC Xena platform. Kaplan-Meier survival analysis showed that expression of NSUN5, but not other NSUN proteins, is positively associated with shorter survival of GBM patients. Median expression value was used as cut-off of high versus low mRNA expression of the NSUN family members. **(B)** Tumor GBM-TCGA-540 dataset (Affymetrix U133A mRNA expression profiling) was analyzed using the scan mode option, which generates the most significant expression cut-off for survival analysis. Kaplan-Meier survival analysis showed that the expression of NSUN5 is significantly associated with shorter survival of GBM patients, whereas the expression of NSUN3 and NSUN6 is significantly associated with longer survival of GBM patients. The expression of NSUN1 is also significantly associated with shorter survival of GBM patients, but to much less extent compared to NSUN5.

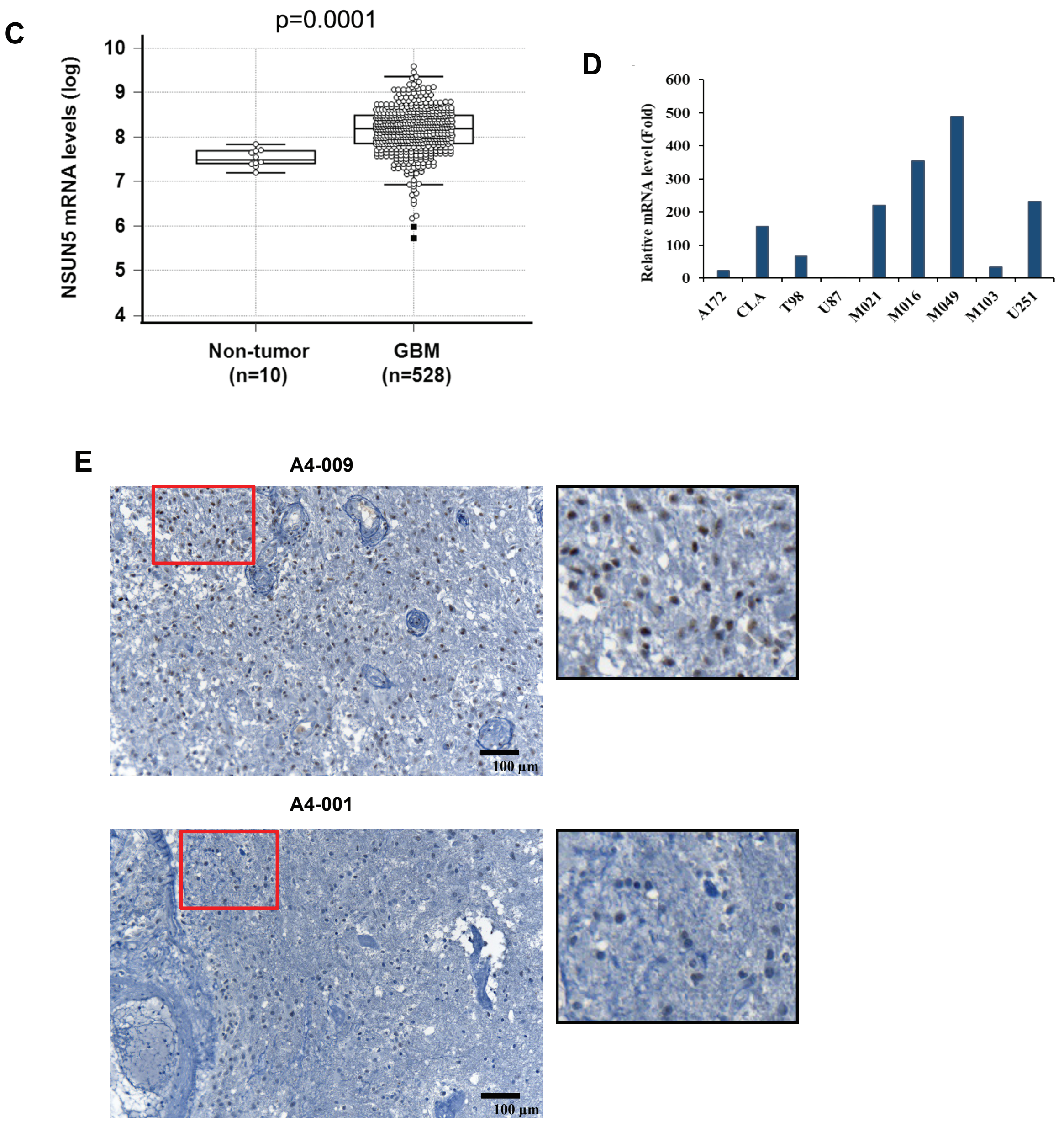

Supplementary Figure 1. Expression of NSUN5 in GBM patients (Cont'd). (C) Phenotype and gene expression data of a GBM cohort (TGCA_GBM HG-U133A) were downloaded from GlioVis platform (http://gliovis.bioinfo.cnio.es/). Independent samples t-test mode of MedCalc (Version 20.210) was employed to test the statistical significance of difference between the NSUN5 mRNA levels of normal brain (non-tumor) and GBM tissues from the same cohort. NSUN5 mRNA values were log-transformed. The analysis showed that NSUN5 mRNA levels are significantly higher in human GBM than non-tumor tissues. (D) NSUN5 mRNA levels were examined by RT-qPCR in 9 GBM cell lines. The expression of NSUN5 is normalized against GAPDH (the internal control) and presented as fold change relative to that of U87 cells that expresses the lowest levels of NSUN5 mRNA. (E) NSUN5 expression in human GBM tissues was examined by immunohistochemistry. Two patient samples with high (A4-009) and low (A4-001) NSUN5 expression are shown. Scale bar = 100 µm. Images in the magnification boxes were enlarged by 2.5-fold and shown on the right.

Original sequence	CGGCCTCCTC CGTGCGGAGA AGAAGCTGCG GCCGCACCTG GCCAAGGTGA GGGGAGGGG AGGGACGGGGA
WTA1	CGGCCTCCTC CGTGCGGAGA AGAAGCTGCG GCCGCACCTG GCCAAGGTGA GGGGAGGGG AGGGACGGGGA
	CGGCCTCCTC CGTGCGGAGA AGAAGCTGCG GCCGCACCTG GCCAAGGTGA GGGGAGGGG AGGGACGGGGA
KOB1	CGGCCTCCTC CGTTGCGGAGA AGAAGCTGCG GCCGCACCTG GCCAAGGTGA GGGGAGGGG AGGGACGGGGA
	CGGCCTCCTC C --- ------ ------ A AGAAGCTGCG GCCGCACCTG GCCAAGGTGA GGGGAGGGG AGGGACGGGGA
	CGGCCTCCTC C ---- ----- ------ A AGAAGCTGCG GCCGCACCTG GCCAAGGTGA GGGGAGGGG AGGGACGGGGA
	CGGCCTCCTC --GTGCGGAGA AGAAGCTGCG GCCGCACCTG GCCAAGGTGA GGGGAGGGG AGGGACGGGGA
	CGGCCTCCTC --GTGCGGAGA AGAAGCTGCG GCCGCACCTG GCCAAGGTGA GGGGAGGGG AGGGACGGGGA
KOB21	CGGCCTCCTC CGTTGCGGAGA AGAAGCTGCG GCCGCACCTG GCCAAGGTGA GGGGAGGGG AGGGACGGGGA
	CGGCCTCCTC CGTTGCGGAGA AGAAGCTGCG GCCGCACCTG GCCAAGGTGA GGGGAGGGG AGGGACGGGGA
	CGGCCTCCTC CGTTGCGGAGA AGAAGCTGCG GCCGCACCTG GCCAAGGTGA GGGGAGGGG AGGGACGGGGA
	CGGCCTCCTC CG ------ ------ ------ ------ ------ ------ GCCGCACCTG GCCAAGGTGA GGGGAGGGG AGGGACGGGGA
	CGGCCTCCTC CG ------ ------ ------ ------ ------ ------ GCCGCACCTG GCCAAGGTGA GGGGAGGGG AGGGACGGGGA
	CGGCCTCCTC AGTGCG--------A AGAAGCTGCA GCCGCACCTG GCCAAGG ---------- GTAGGGG CGGGGCGGGGA
KOB22	CGGCCTCCTC CGTTGCGGAGA AGAAGCTGCG GCCGCACCTG GCCAAGGTGA GGGGAGGGG AGGGACGGGGA
	CGGCCTCCTC CGTTGCGGAGA AGAAGCTGCG GCCGCACCTG GCCAAGGTGA GGGGAGGGG AGGGACGGGGA
	CGGCCTCCTC ----TGCGGAGA AGAAGCTGCG GCCGCACCTG GCCAAGGTGA GGGGAGGGG AGGGACGGGGA
	CGGCCTCCTC AGTGCG------A AGAAGCTGCA GCCGCACCTG GCCAAGG ---------- GTAGGGG CGGGGCGGGGA
	CGGCCTCCTC AGTGCG------A AGAAGCTGCA GCCGCACCTG GCCAAGG ---------- GTAGGGG CGGGGCGGGGA
	CGGCCTCCTC AGTGCG------A AGAAGCTGCA GCCGCACCTG GCCAAGG ---------- GTAGGGG CGGGGCGGGGA

Supplementary Figure 2. Genomic DNA sequencing of U251 NSUN5 KO clones. Sanger sequencing of the genomic DNA confirmed generation of indels in the exon II of the NSUN5 gene by CRISPR editing in NSUN5 KO clones. WA1, the wild-type control, showed normal DNA sequence. Mutations in NSUN5 KO clone KOB1 (1-nucleotide insertion, 1-nucleotide deletion, and 8-nucleotide deletion) and KOB22 (1-nucleotide insertion, 2-nucleotide deletion, and 8-nucleotide deletion) were frameshift mutations, which explains loss of NSUN5 protein expression in these two clones. Mutations in KOB21 (1-nucleotide insertion, 4-nucleotide deletion, and 18-nucleotide deletion) caused frameshift in one allele and deletion of 6 amino acids in another allele of the NSUN5 gene, which explains the presence of a weak and smaller NSUN5 band detected Western blotting.

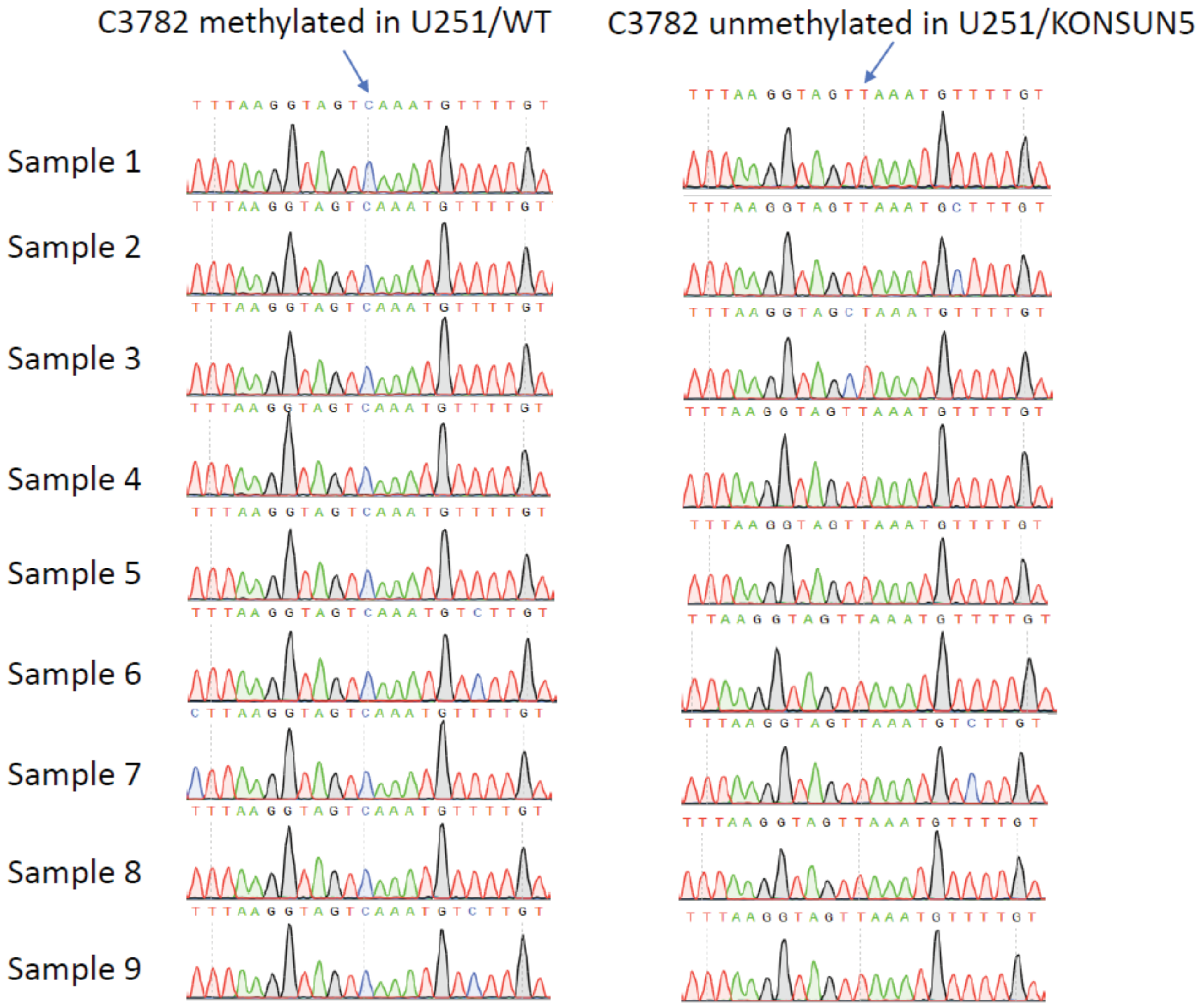

Supplementary Figure 3. Sanger sequencing chromatograms of the bisulfite sequencing results of U251 WT and KO cells. RNAs isolated from U251 WT and KO cells were used for bisulfite sequencing to determine the methylation status of cytosine 3782 (C3782) of 28 rRNA. Sanger sequencing chromatograms of the bisulfite sequencing shown here illustrate that C3782 is methylated in U251 WT, but not in U251 KO cells. The results were summarized and shown in Fig. 2B.

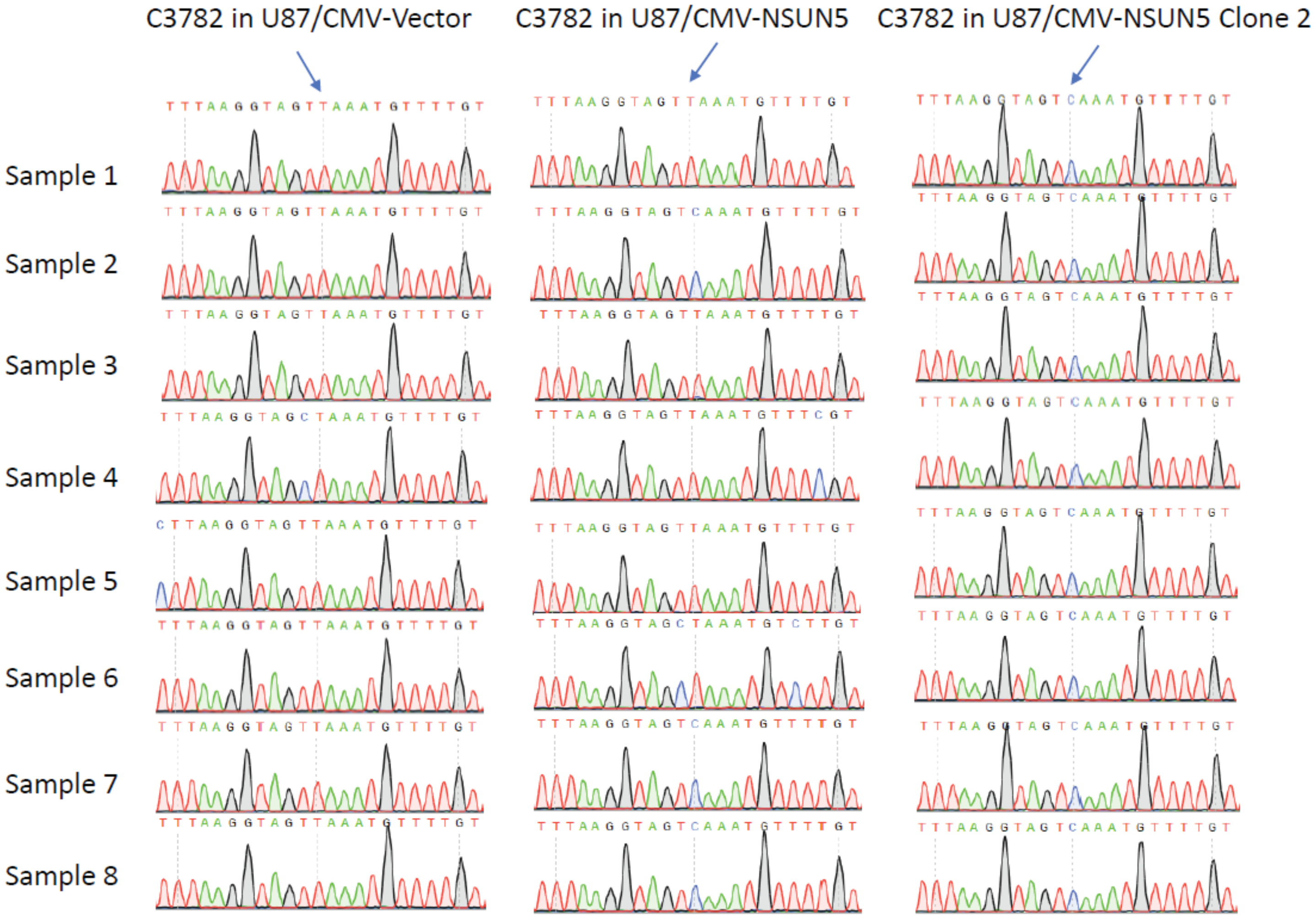

Supplementary Figure 4. Sanger sequencing chromatograms of the bisulfite sequencing results of U87 cells. RNAs isolated from U87 cells stably transfected with empty vector or NSUN5 were used for bisulfite sequencing to determine the methylation status of cytosine 3782 (C3782) of 28 rRNA. Sanger sequencing chromatograms of the bisulfite sequencing shown here illustrate that C3782 was not methylated in U87 cells transfected with empty vector but was methylated in U87 cells transfected with NSUN5. These results indicate that C3782 methylation is dependent on the presence of NSUN5 in U87 cells. The results were summarized and shown in Fig. 2D.

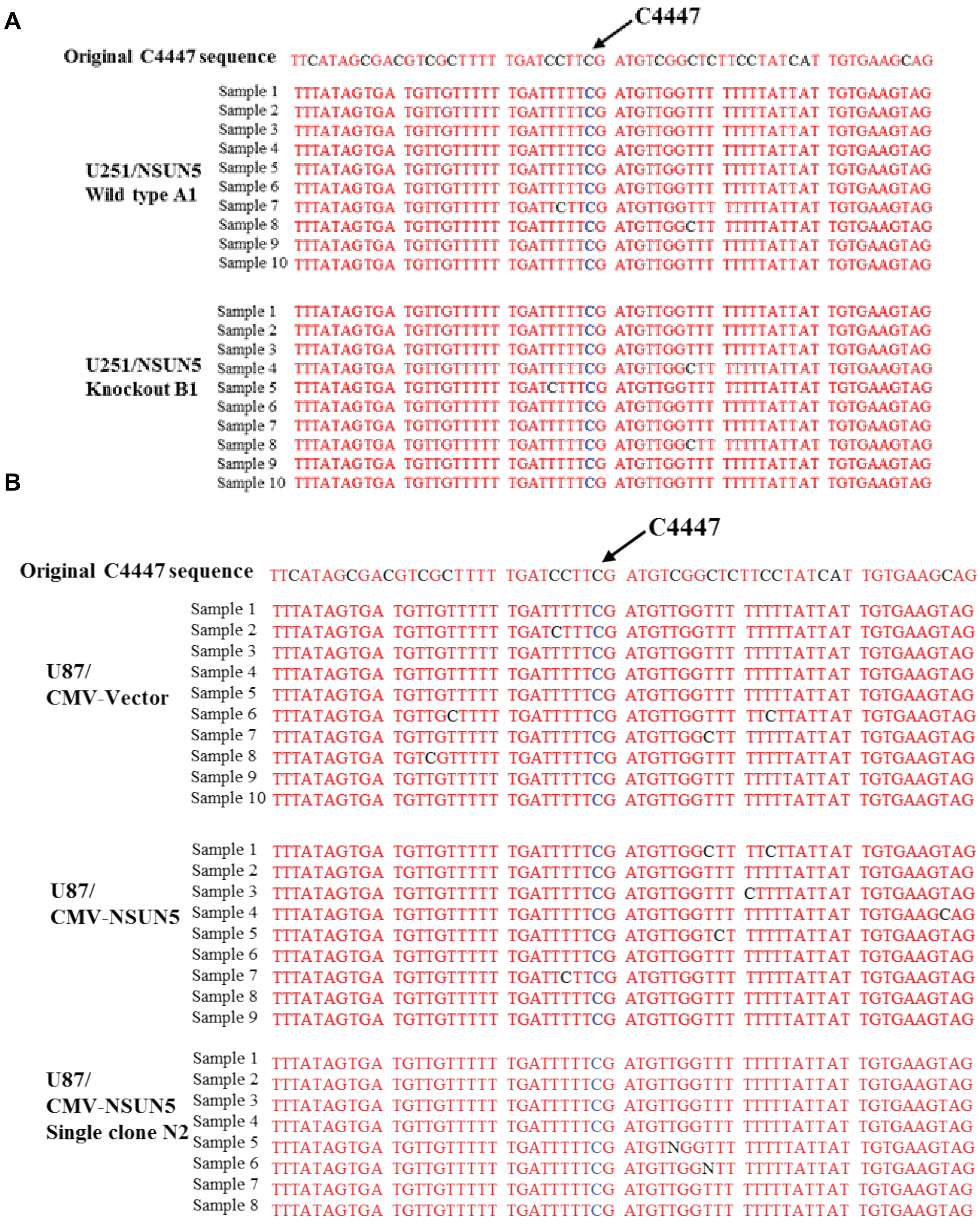

Supplementary Figure 5. NSUN1-mediated C4447 methylation of 28S rRNA was not affected by NSUN5 KO in U251 or overexpression in U87 cells. RNAs isolated from U251 WT and NSUN5 KO cells, as well as U87 cells stably transfected with empty vector or NSUN5 were used for bisulfite sequencing to determine the methylation status of cytosine 444 (C444) of 28 rRNA. Bisulfite sequencing results showed that the methylation status of C4444 of 28 rRNA, which is induced by NSUN1, was not affected by NSUN5 knockout in U251 cells or by NSUN5 overexpression in U87 cells.

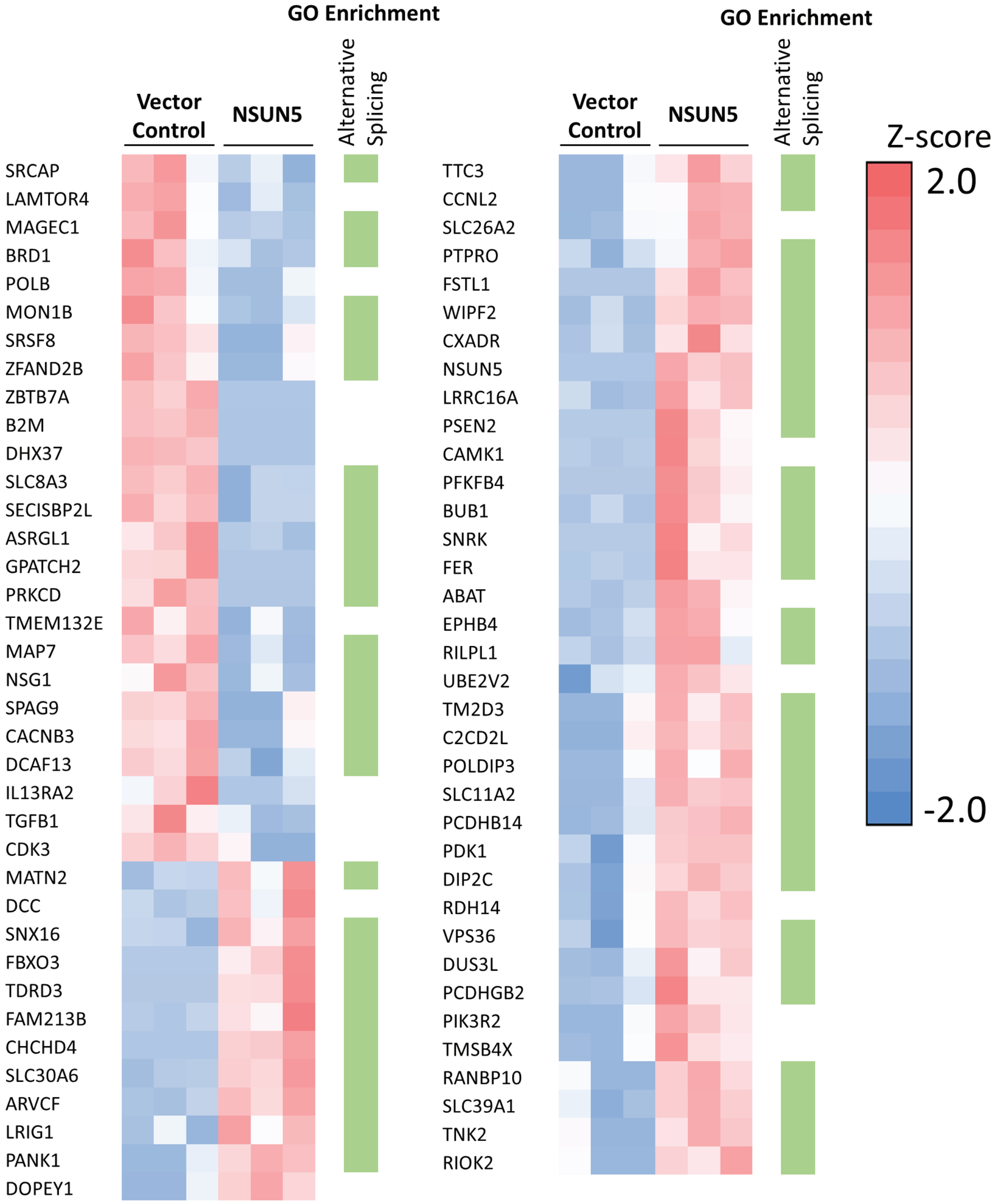

Supplementary Figure 6. Heatmap of the proteins altered by NSUN5 overexpression in 50M cells. Heatmap of the proteins identified to be significantly ($p<0.05$) changed in expression by LFQ proteomic analysis upon NSUN5 overexpression versus empty vector control in 50M cells. Cluster alignment of the data was performed with Clustergrammer. Columns represent quantified data from individual samples. Green boxes indicate the proteins that were identified in GO enrichment analysis.

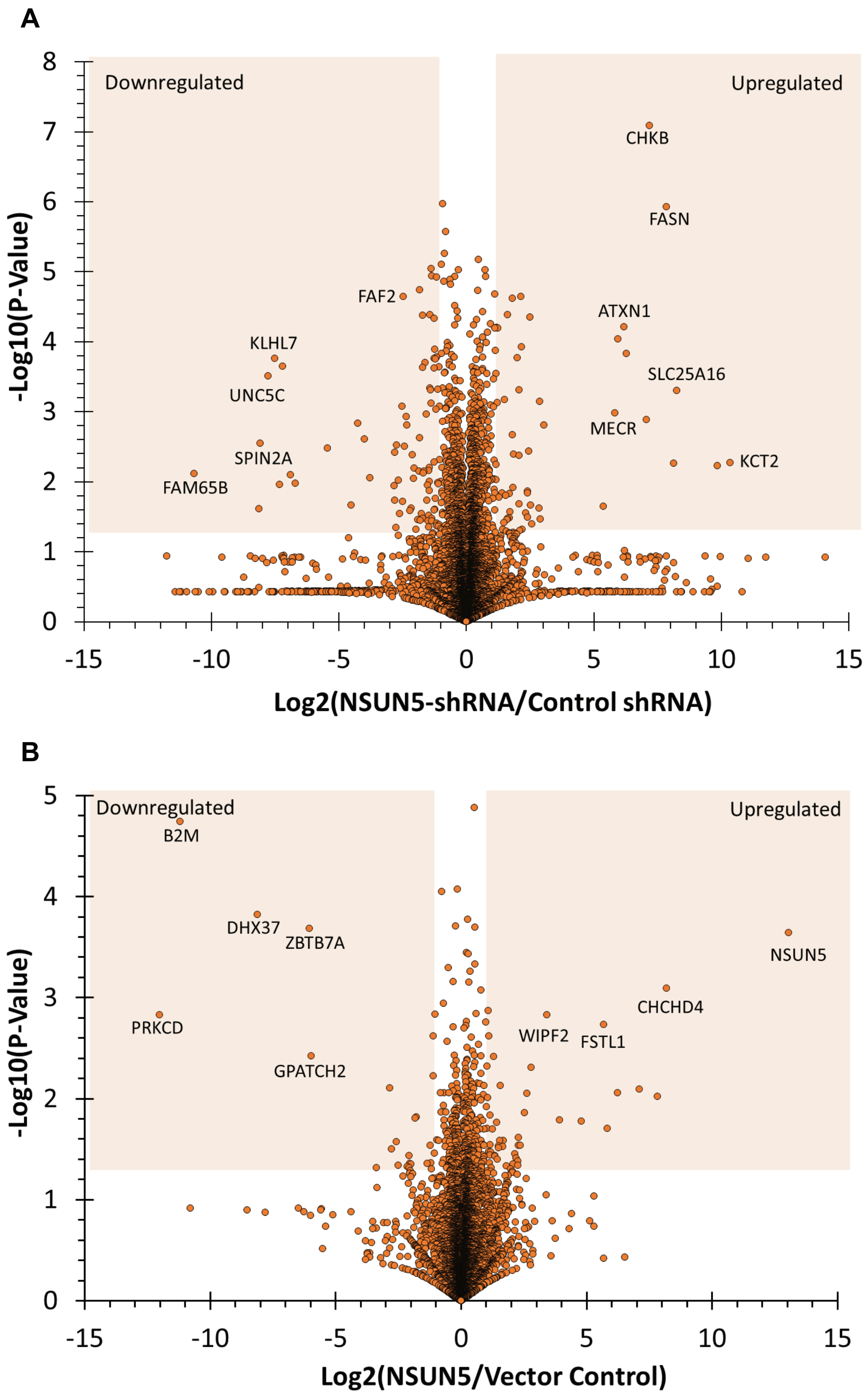

Supplementary Figure 7. Volcano plots of the proteins altered by NSUN5 knockdown in U251 (A) and NSUN5 overexpression in 50M cells (B). Proteins that are altered by at least 2-fold and have p value less than 0.05 ($p<0.05$) are shown in the shaded area. Some proteins are labeled with their names.

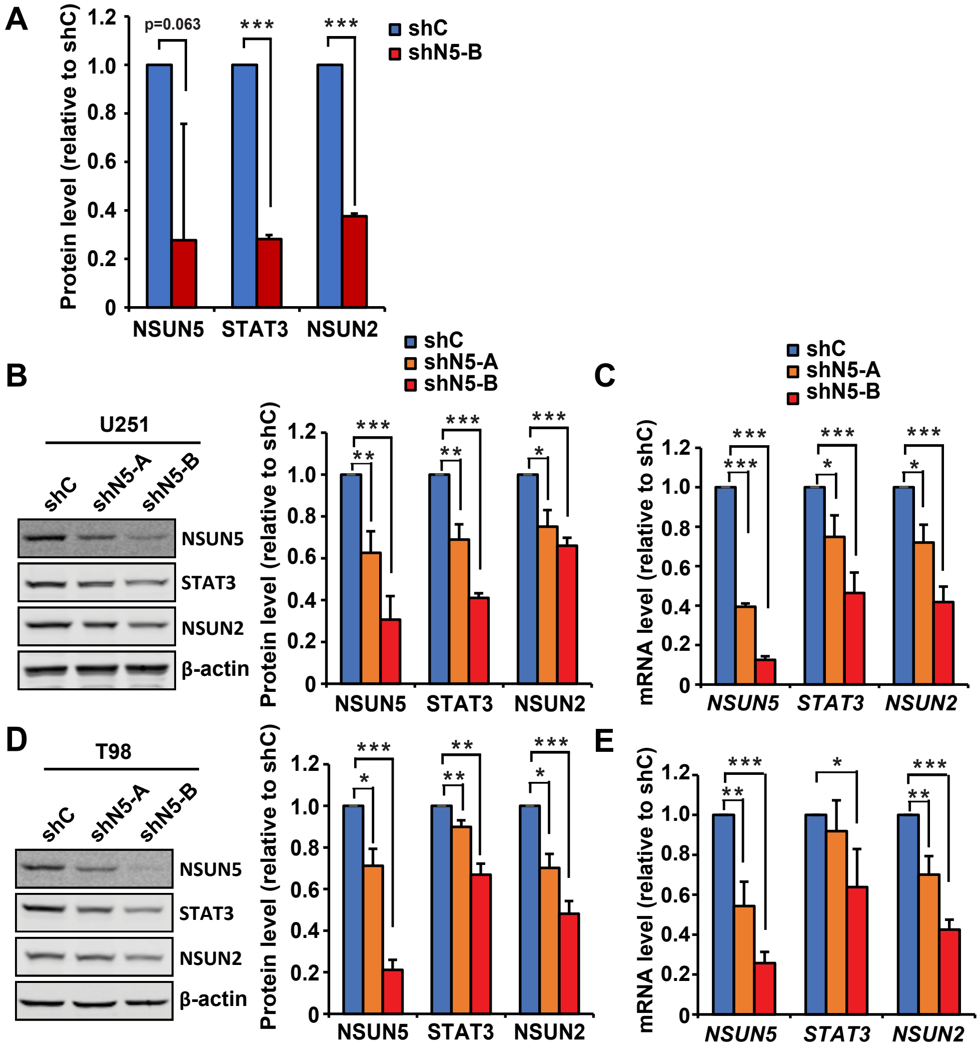

Supplementary Figure 8. NSUN5 knockdown leads to decreased expression of STAT3 and NSUN2. **(A)** LC-MS/MS showed that NSUN5, STAT3, and NSUN2 protein levels were decreased in U251/shNSUN5-B cells compared U251/shControl cells. Data are mean ± SD of three replicates. **(B** and **D)** Western blotting showed that NSUN5 knockdown leads to decreased protein level of STAT3 and NSUN2 in U251 cells and T98 cells. β-actin was used as the loading control. Protein levels were quantified by standardizing against the loading control and expressed as a relative value with those in shC set as 1. **(C** and **E)** RT-qPCR data showed that NSUN5 knockdown leads to decreased mRNA levels of STAT3 and NSUN2 in U251 and T98 cells. mRNA levels were quantified by standardizing against GAPDH control and expressed as a relative value with those in shC set as 1. Data are mean ± SE of three independent experiments. Significantly different (Student's t-est, * $p < 0.05$, ** $p < 0.01$, *** $p < 0.001$). shC: shControl; shN5-A: shNSUN5-A; shN5-B: shNSUN5-B.

A **U251 cells**

Variable Y	NSUN5_P
Variable X	NSUN5_R

Sample size	15
Spearman's coefficient of rank correlation	0.800
Significance level	P=0.0003
95% Confidence Interval for rho	0.488 to 0.931

Variable Y	STAT3_P
Variable X	STAT3_R

Sample size	15
Spearman's coefficient of rank correlation	0.744
Significance level	P=0.0015
95% Confidence Interval for rho	0.375 to 0.910

Variable Y	NSUN2_P
Variable X	NSUN2_R

Sample size	15
Spearman's coefficient of rank correlation	0.593
Significance level	P=0.0199
95% Confidence Interval for rho	0.115 to 0.848

B **T98 cells**

Variable Y	NSUN5_P
Variable X	NSUN5_R

Sample size	9
Spearman's coefficient of rank correlation	0.983
Significance level	P<0.0001
95% Confidence Interval for rho	0.917 to 0.996

Variable Y	STAT3_P
Variable X	STAT3_R

Sample size	9
Spearman's coefficient of rank correlation	0.776
Significance level	P=0.0140
95% Confidence Interval for rho	0.231 to 0.950

Variable Y	NSUN2_P
Variable X	NSUN2_R

Sample size	9
Spearman's coefficient of rank correlation	0.862
Significance level	P=0.0028
95% Confidence Interval for rho	0.463 to 0.971

Supplementary Figure 9. Levels of mRNA and protein are significantly correlated for NSUN5, ATAT3 and NSUN2. Spearman's correlation between the levels of mRNA (NSUN5_R, STAT3_R, NSUN2_R) and protein (NSUN5_P, STAT3_P, NSUN2_P) was analyzed for each gene, which showed that they are significantly associated for all three genes in both U251 **(A)** and T98 **(B)** cells.

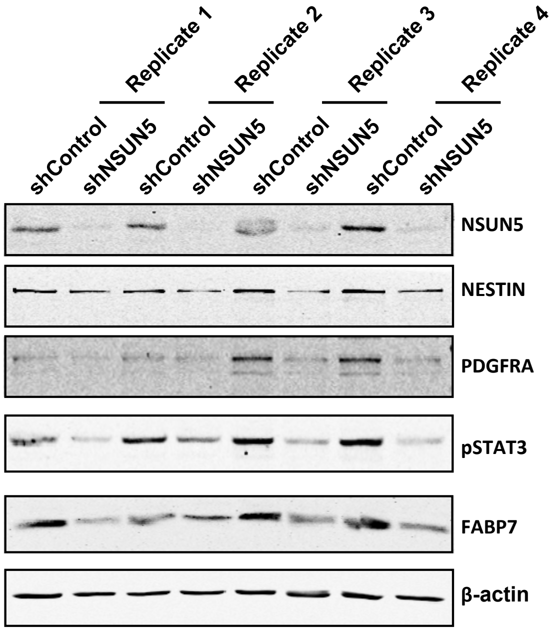

Supplementary Figure 10. NSUN5 knockdown leads to decreased protein level of some key factors in GBM. Cell lysates were prepared from four different batch of U251/shControl and U251/shNSUN5 cells for Western blotting validation the proteins identified in the proteomic analysis. Western blotting showed that some key factors in GBM, including Nestin, PDGFRA, pSTAT3, and FABP7 were downregulated when NSUN5 was knocked down in U251 cells. β-actin was used as the loading control.

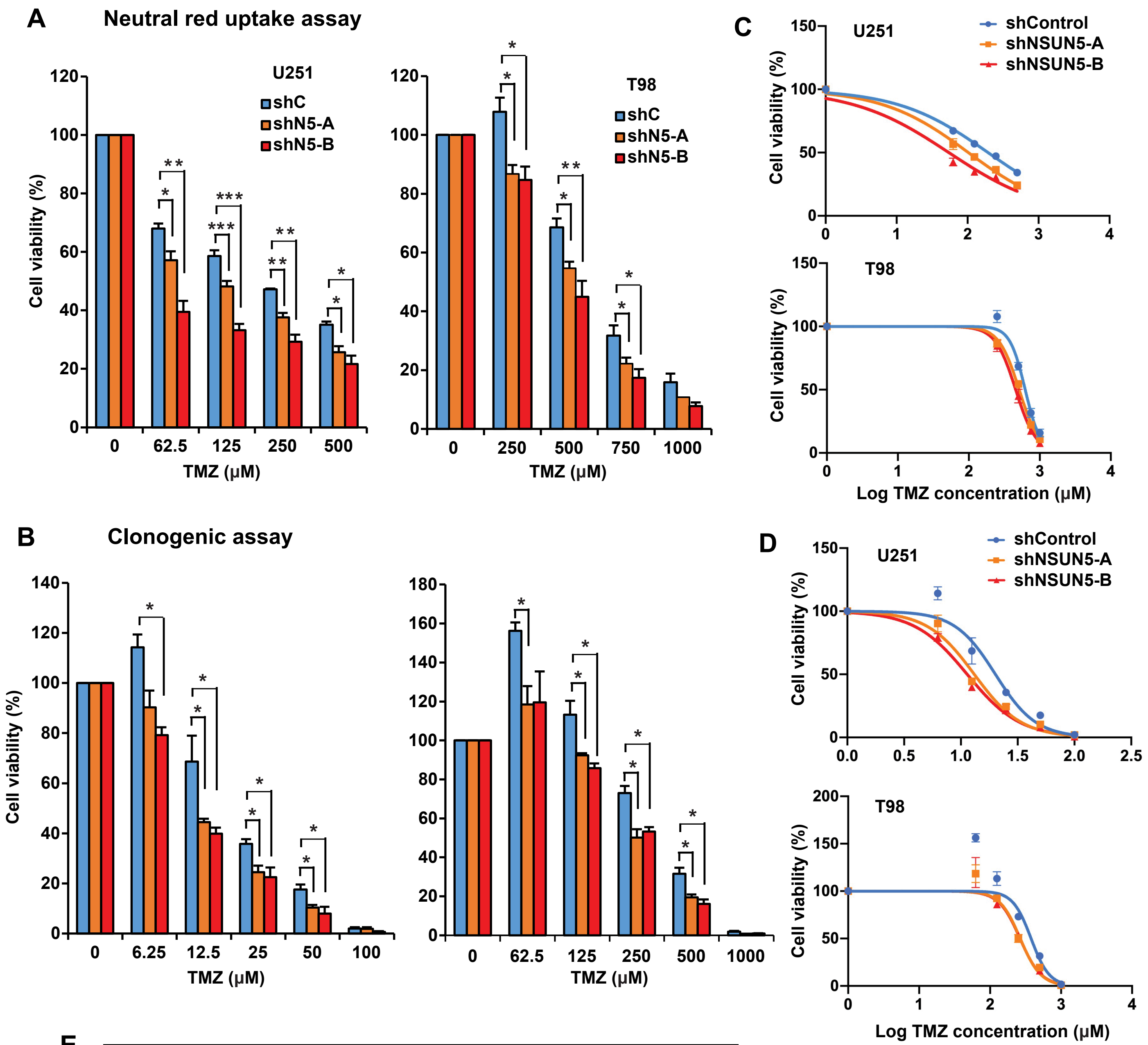

E

Cells	Neutral red uptake assay		Clonogenic assay	
	U251	T98	U251	T98
shControl	194.5	623.3	20.2	378.2
shNSUN5-A	102.7	512.3	13.1	366.1
shNSUN5-B	54.5	457.6	11.4	264.4

Supplementary Figure 11. Knockdown of NSUN5 sensitizes GBM cells to temozolomide. U251 and T98 cells infected with lentivirus expressing shControl, shNSUN5-A, or shNSUN5-B were treated with increasing doses of temozolomide (TMZ). Cell viability was determined by the neutral red uptake assay **(A)** and clonogenic assay **(B)**. Data are shown as mean ± SE of three independent experiments. Significant different (Student's t-test, * $p < 0.05$, ** $p < 0.01$, *** $p < 0.001$). IC50 was calculated **(C** and **D)** and shown in a table **(E)**. shC: shControl; shN5-A: shNSUN5-A; shN5-B: shNSUN5-B.

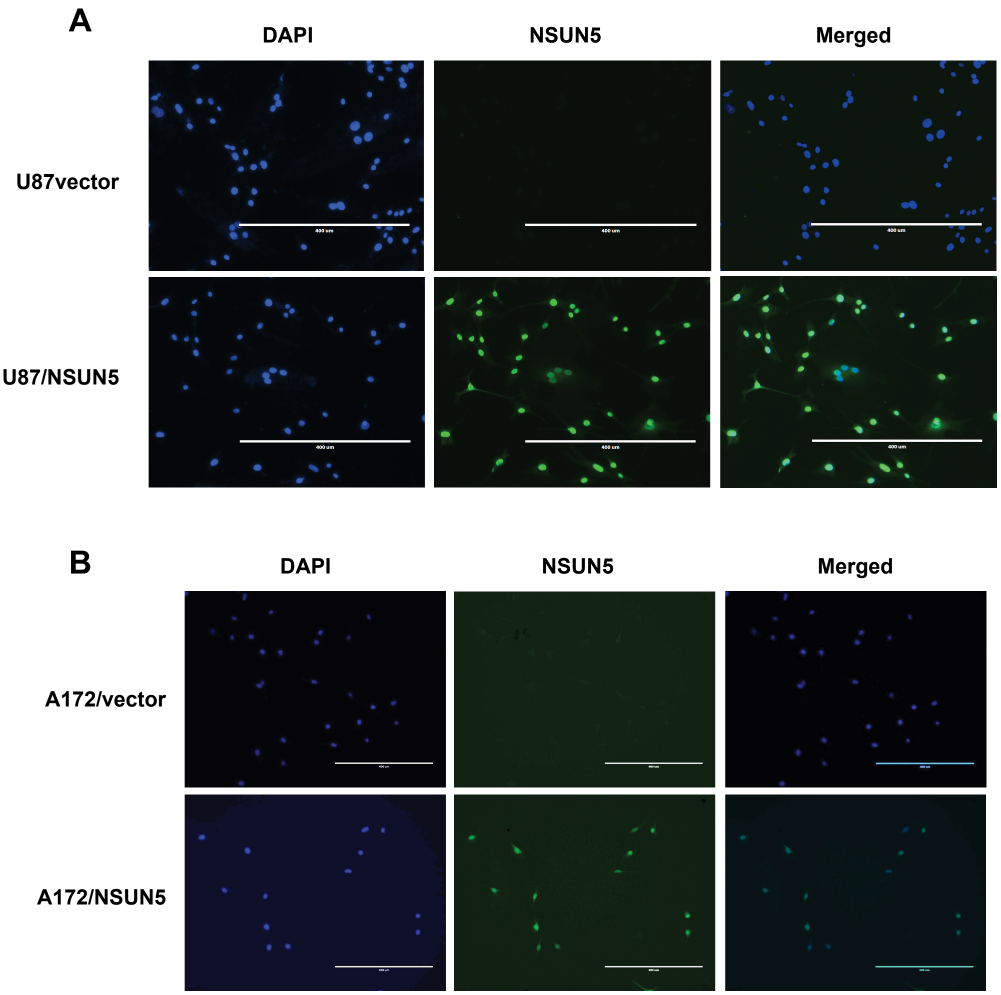

Supplementary Figure 12. Immunocytochemistry of the overexpressed NSUN5 in U87 and A172 cells. U87 and A172 cells were stably transduced with pLenti-vector or pLenti-NSUN5. Overexpression of NSUN5 in U87 **(A)** and A172 **(B)** cells was confirmed by immunocytochemistry using an antibody against NSUN5. DAPI was used to stain the nucleus. Scale bar = 400 μm.

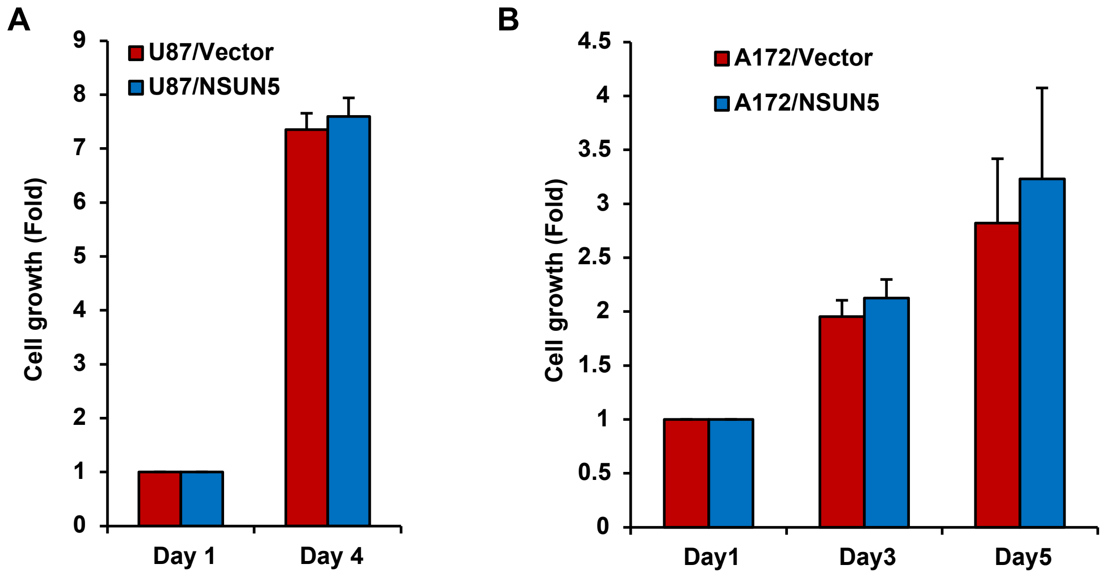

Supplementary Figure 13. Overexpression of NSUN5 does not affect cell growth in U87 and A172 cells cultured in the adherent conditions. U87 **(A)** and A172 **(B)** cells stably expressing an empty vector or NSUN5 were seeded into 96-well plates. Cell growth was measured at day 4 for U87 cells and day 3 and day 5 for A172 cells using the neutral red uptake assay and expressed as the fold change relative to day 1. Data are mean ± SD of three independent experiments.

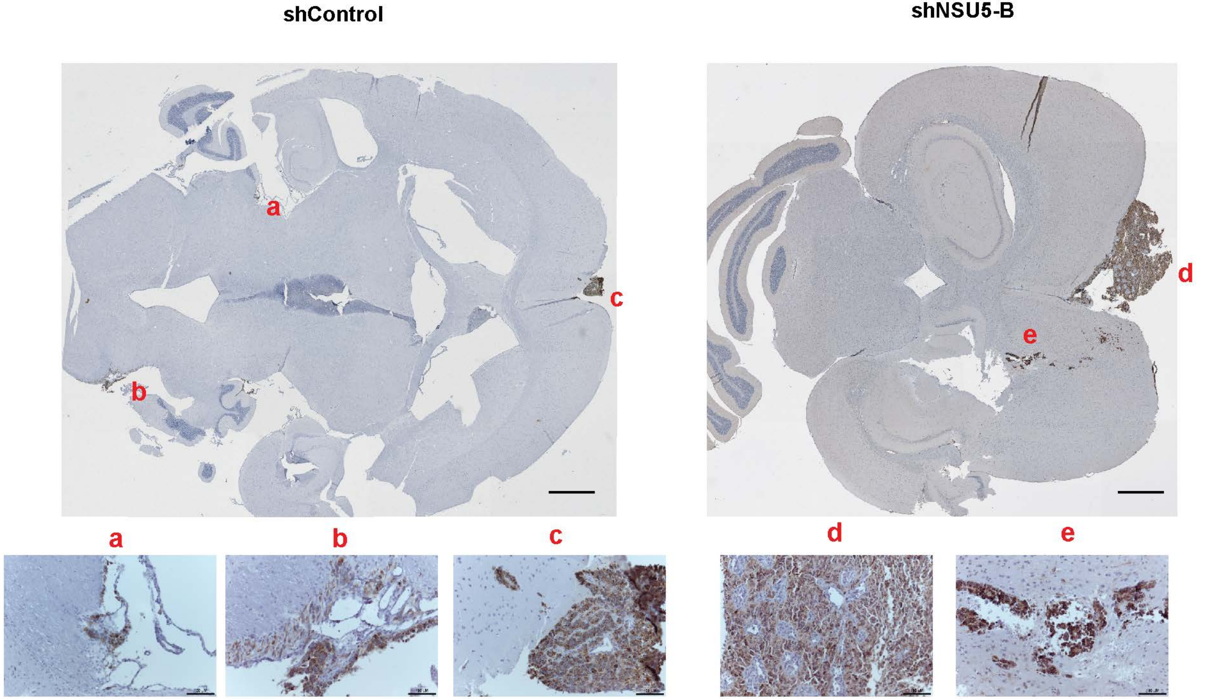

Supplementary Figure 14. U251 tumors in the brains of the mice. U251 cells stably transfected with shControl and shNSUN5-B were intracranially injected into NSG mice. After euthanasia, brains were collected from the mice and U251 tumors were detected by immunohistochemistry using an antibody that specifically recognize a human mitochondrial protein. The top panel is the IHC image of the section of whole brain generated using tile imaging, which detected sites of tumor cells. Scale bar = 1 mm. The bottom are the IHC image of high magnification of the tumors identified in the top panel as labelled by the letters (a to e). Scale bar = 100 μm.

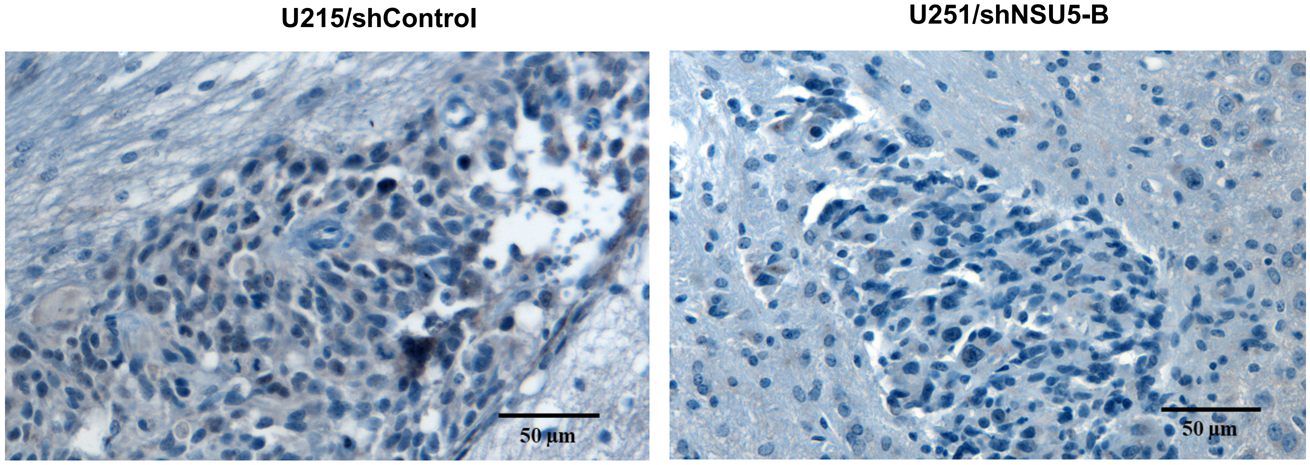

Supplementary Figure 15. Expression of NSUN5 in U251/shControl and U251/shNSUN5 tumors. Expression of NSUN5 in U251/shControl and U251/shNSUN5 tumors were examined by IHC using an anti-NSUN5 antibody. The results showed that NSUN5 was expressed in NSUN5 in U251/shControl tumors, but not in U251/shNSUN5 tumors, confirming knockdown of NSUN5 in U251/shNSUN5 tumors. Scale bar = 50 µm

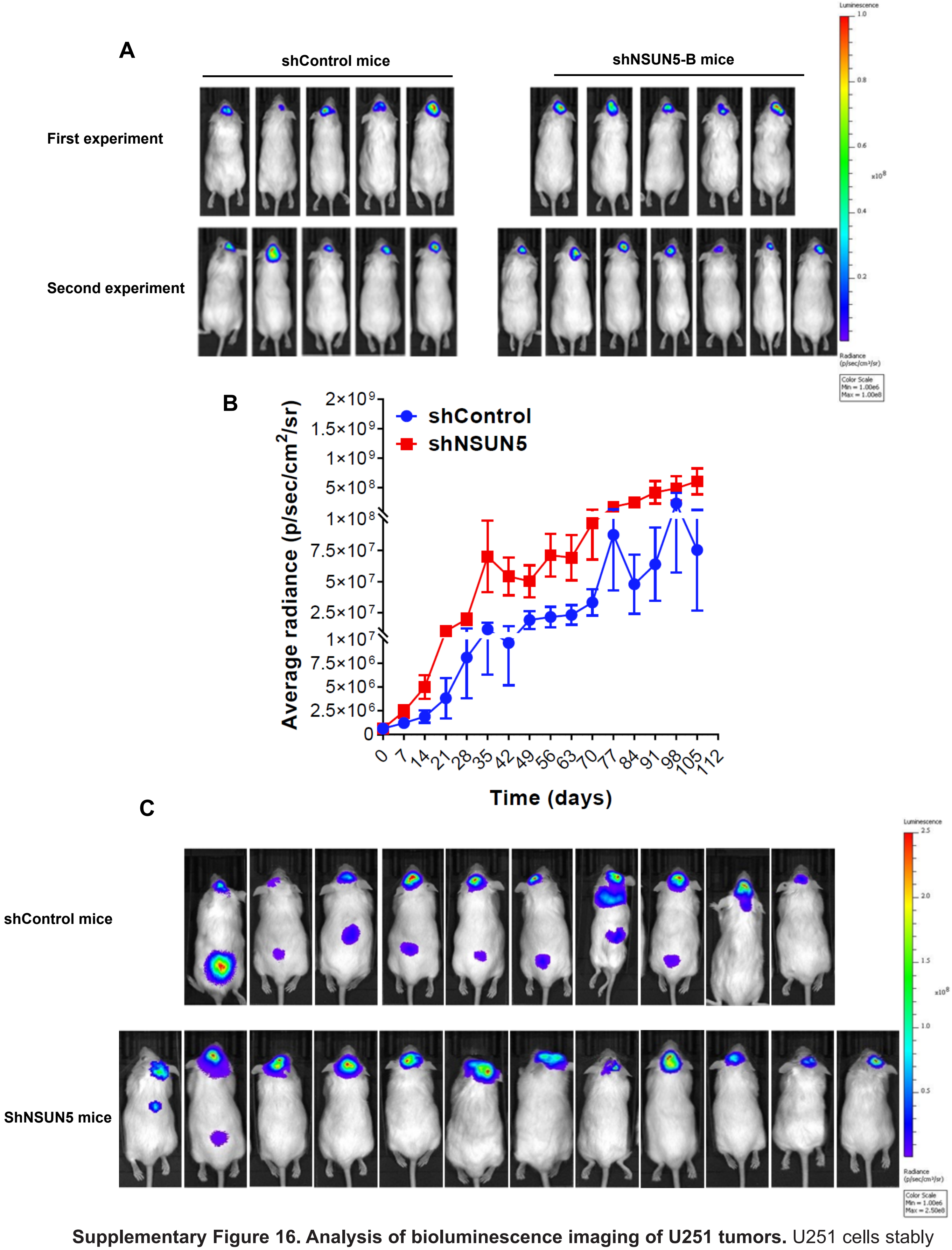

Supplementary Figure 16. Analysis of bioluminescence imaging of U251 tumors. U251 cells stably transfected with shControl and shNSUN5-B were intracranially injected into NSG mice. Tumor formation and progression were monitored by bioluminescence imaging (BLI). **(A)** BLI of the intracranial tumors at day 21. **(B)** Tumor formation and progression were monitor by BLI and expressed as average radiance. **(C)** Tumors detected by BLI in the spinal cord in 8 out of 10 mice bearing U251/shControl tumors and in 2 out of 12 mice bearing U251/shNSUN5-B tumors.

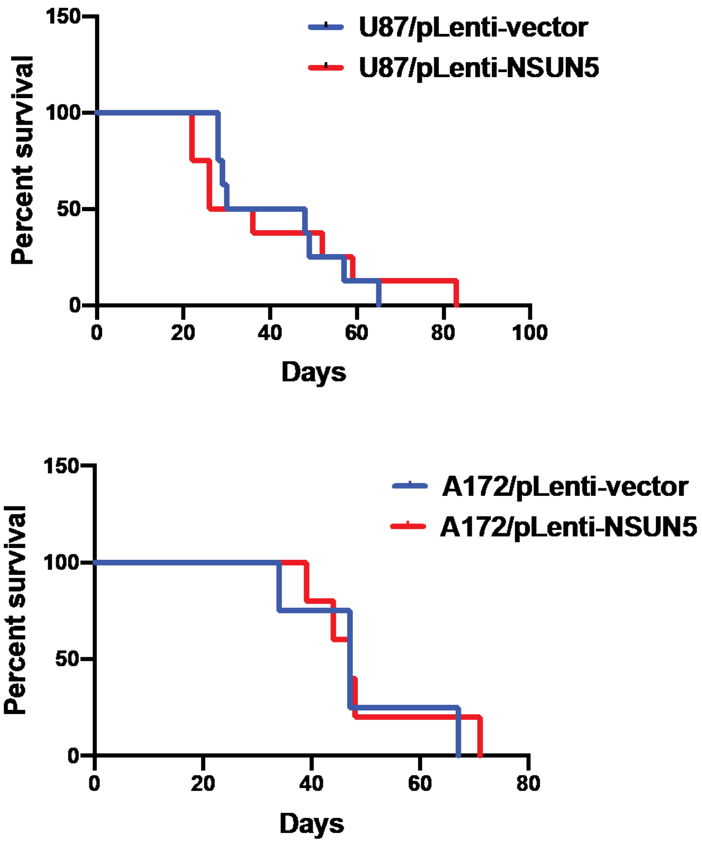

Supplementary Figure 17. Overexpression of NSUN5 did not change the survival of mice bearing U87 and A172 tumors. U87/Vector and U87/NSUN5 cells, as well as, A172/Vector and A172/NSUN5 cells were intracranially injected into NSG mice. Survival of mice bearing the tumors was recorded. Overexpression of NSUN5 in U87 and A172 cells did not change the survival of mice bearing the intracranial tumors (n = 8 for U87/vector tumors; n = 8 for U87/NSUN5 tumors; n = 4 for A172/vector tumors; and n =5 for A172/NSUN5 tumors)

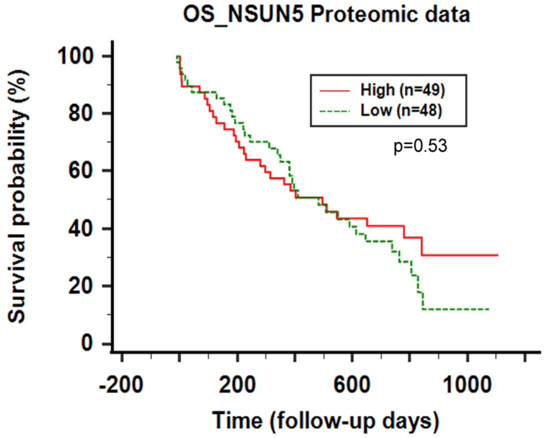

Supplementary Figure 18. Association between NSUN5 protein level and survial of GBM patients. NSUN5 protein levels and patient survival are not associated in the dataset published by Liang-Bo Wang et al. (Cancer Cell. 2021 Apr 12;39(4):509-528.e20). The median value of normalized protein levels (-0.012) was used as the cut-off to stratify NSUN5-high vs NSUN5-low patient populations.
