# Supplementary material for: RNA cytosine methyltransferase NSUN5 promotes protein synthesis and tumorigenic phenotypes in glioblastoma
Source: Mol Oncol. 2023 Apr 22;17(9):1763–83. doi: 10.1002/1878-0261.13434 (PMC10483612; doi:10.1002/1878-0261.13434)
Supplement: Supplementary file 2 — Table S1. Sequencing of gRNAs, shRNAs, and PCR primers. [file MOL2-17-1763-s002.docx]

Supplementary Table S1. Sequencing of gRNAs, shRNAs, and PCR primers

| NSUN5 CRISPR guide RNA 1 | 5’GCGCCGGCCTCCTCCGTG3’ (Exon II, sense) | |
| --- | --- | --- |
| NSUN5 CRISPR guide RNA 2 | 5’GAGCTTCTTCTCCGCACGG3’ (Exon II, antisense) | |
| shNSUN5-A | 5’AGACCACACTCAGCAGTGGCTTCTTCGTT3’ | |
| shNSUN5-B | 5’GGCCAAGGTGCTAGTGTATGAGTTGTTGT3’ | |
| PCR primers | Forward | Reverse |
| Bisulfite sequencing primers for cytosine 3872 of 28s rRNA | 5'TGTGGGTAAATGGTGGGAGTA3' | 5'AACACCAAAAACCTCCCACCTA3’ |
| Bisulfite sequencing primers for cytosine 4447 of 28s rRNA | 5'GATTGTGAAAGTGGGGTTTTATG3’ | 5'AAACCCAACTCACATTCCCTATT3' |
| NSUN5 | 5'CTTCTGAAGAACCAAGGGAAGA3' | 5'AGCCAGTTCACAGCAAGAG3' |
| STAT3 | 5’GAGAAGGACATCAGCGGTAAG3’ | 5’CGATGGAGACACCAGGATATT3’ |
| NSUN2 | 5’GTGGAATAAACGTCAGCCAAA3’ | 5’CTTAGAGGGATCTGTGGGTTTC3’ |
| GAPDH | 5'GGACCTGACCTGCCGTCTAGAA3' | 5'GGTGTCGCTGTTGAAGTCAGAG3' |
