# Supplementary material for: RNA cytosine methyltransferase NSUN5 promotes protein synthesis and tumorigenic phenotypes in glioblastoma
Source: Mol Oncol. 2023 Apr 22;17(9):1763–83. doi: 10.1002/1878-0261.13434 (PMC10483612; doi:10.1002/1878-0261.13434)
Supplement: Supplementary file 3 — Table S2. Instrument parameters for data acquisition. [file MOL2-17-1763-s003.docx]

Supplemental Table S2: Instrument parameters for data acquisition

| **QE Plus Setting** | **Value** |
| --- | --- |
| Scan Range | 400-1500 m/z |
| MS1 AGC Target | 3E6 |
| MS1 Resolution | 70K |
| MS2 Resolution | 17.5K |
| MS2 AGC Target | 2E5 |
| Maximum IT | 64ms |
| Loop Count | 12 |
| Top N | 12 |
| Isolation Window | 1.2 m/z |
| Isolation Offset | 0.5 m/z |
| NCE | 25 |
| Dynamix Exclusion | 30 |
| Charge Exclusion | 1,7,8,>8 |
| Fixed First Mass | 100 m/z |
